# Schizophrenia interactome: fully-labeled interactome network

**DOI:** 10.1038/npjschz.2016.25

**Published:** 2016-08-24

**Authors:** Madhavi Ganapathiraju, Srilakshmi Chaparala

**Affiliations:** 1 Department of Biomedical Informatics, University of Pittsburgh, Pittsburgh, PA, USA

We recently published the schizophrenia interactome^1^ that showed hundreds of protein-protein interactions (PPIs) of schizophrenia-associated genes, including 504 novel PPIs. Figure 2 in that article gave an overview of how many known or novel PPIs each of the schizophrenia genes had, while the specific interactions were made available through a searchable web-application called Schizo-Pi (http://severus.dbmi.pitt.edu/schizo-pi). Here, we are releasing the interactome figure in which all the nodes (schizophrenia-associated genes and their interactors) and edges (PPIs) are all labeled by their gene symbols, and are color coded by whether they are known or novel PPIs/interactors (Figure 1). An electronically searchable figure is available online as Supplementary File 1 and a list of genes and PPIs in the interactome as Supplementary File 2.

## Figures and Tables

**Figure 1:**
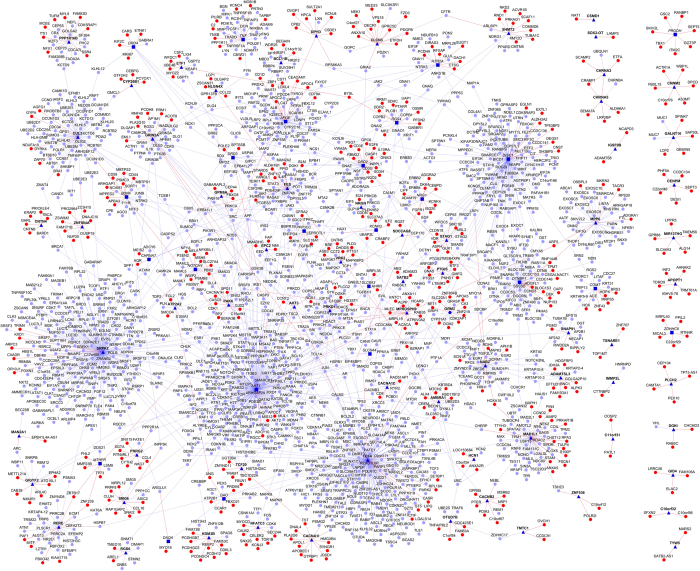
Schizophrenia interactome: Network view of the schizophrenia interactome is shown as a graph,where genes are shown as nodes and PPIs as edges connecting the nodes. Schizophrenia-associated genes are shown as dark blue nodes, novel interactors as red color nodes and known interactors as blue color nodes. The source of the schizophrenia genes is indicated by its label font, where Historic sghenowesn a irtea licized, GWAS genes are shown in bold, and the one gene that is common to both is shown in italicized and bold. For clarity, the source is also indicated by the shape of the node (triangular for GWAS and square for Historic and hexagonal for both). Red edges are the novel interactions, whereas blue edges are known interactions. GWAS, genome-wide association studies of schizophrenia; PPI, protein–protein interaction.
