# Supplementary figures and images for: Schizophrenia interactome: fully-labeled interactome network
Source: NPJ Schizophr. 2016 Aug 24;2:16025–. doi: 10.1038/npjschz.2016.25 (PMC5007984; doi:10.1038/npjschz.2016.25)

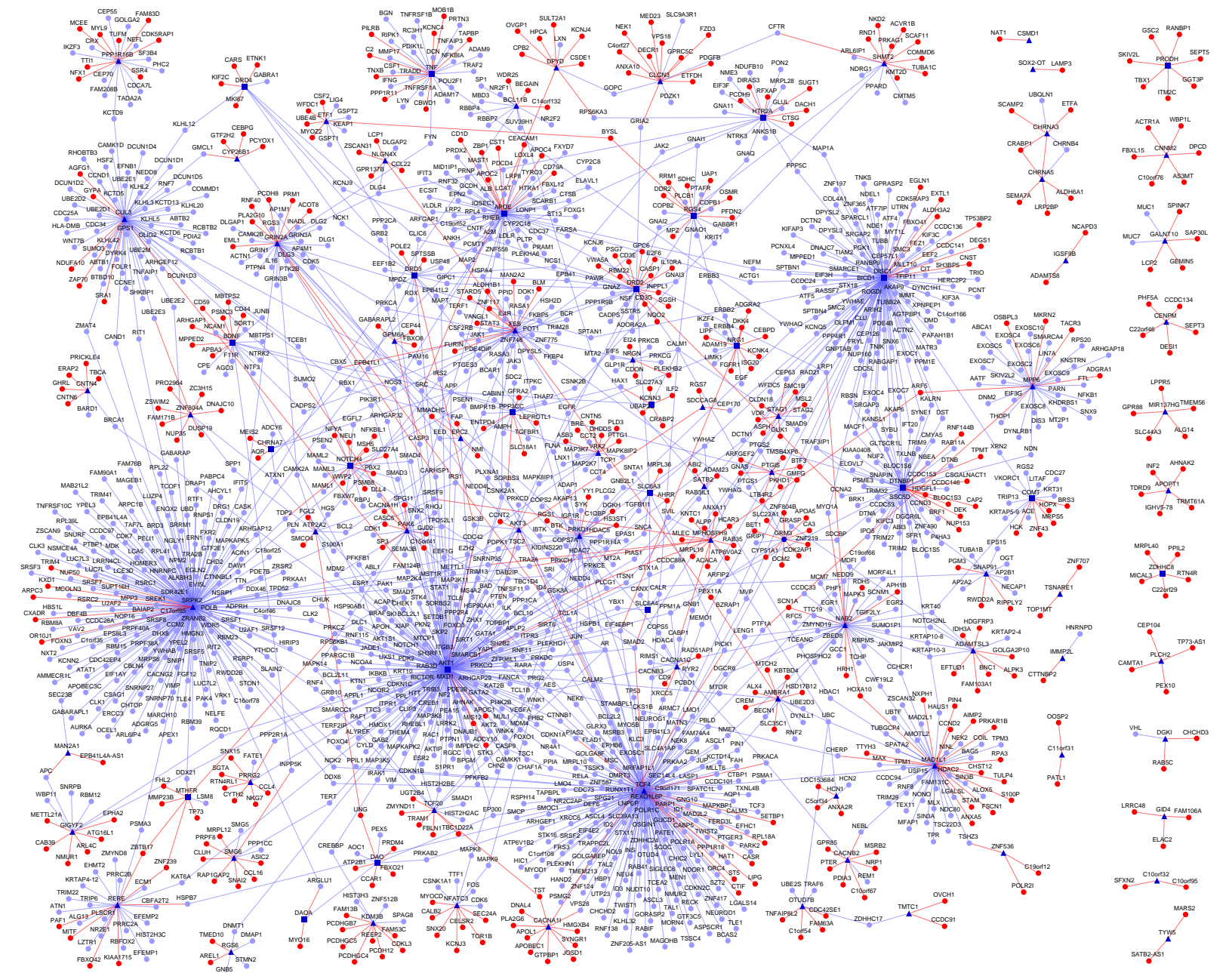

Supplement: Supplementary File 1 [file npjschz201625-s1.pdf]
