# Supplementary material for: Schizophrenia interactome: fully-labeled interactome network
Source: NPJ Schizophr. 2016 Aug 24;2:16025–. doi: 10.1038/npjschz.2016.25 (PMC5007984; doi:10.1038/npjschz.2016.25)
Supplement: Supplementary File 2 [file npjschz201625-s2.pdf]

Schizophrenia Interactome

Ganapathiraju et al, npj Schizophrenia, 2 (16012) 2016. (doi:10.1038/npjschz.2016.12)

Gene 1	Gene 2	Interaction
ABI2	SATB2	Novel
ACACA	MPHOSPH9	Novel
ACOT8	GRIN2A	Novel
ACTR1A	CNNM2	Novel
ACVR1B	SHMT2	Novel
ADAM23	SATB2	Novel
ADAMTS8	IGSF9B	Novel
ADAMTSL3	ALPK3	Novel
ADAMTSL3	BNC1	Novel
ADAMTSL3	EFTUD1	Novel
ADAMTSL3	FAM103A1	Novel
ADAMTSL3	GOLGA2P10	Novel
ADAMTSL3	HDGFRP3	Novel
ADAMTSL3	IDH3A	Novel
ADCY10	AKT1	Novel
ADGRA1	MPP6	Novel
ADGRA2	NRG1	Novel
AGFG1	CUL3	Novel
AHNAK2	APOPT1	Novel
AHRR	SLC6A3	Novel
AIMP2	MAD1L1	Novel
AKT1	APOC1	Novel
AKT1	CDC42	Novel
AKT1	ITSN1	Novel
AKT1	MIS12	Novel
AKT3	CALM2	Novel
AKT3	CCNT2	Novel
AKT3	PTEN	Novel
AKT3	SDCCAG8	Novel
ALDH1B1	FES	Novel
ALDH3A2	DISC1	Novel
ALDH6A1	CHRNA5	Novel
ALG14	MIR137HG	Novel
ALPP	MPHOSPH9	Novel
ALX4	AMBRA1	Novel
AMBRA1	CREM	Novel
AMBRA1	HSD17B12	Novel
AMBRA1	KBTBD4	Novel
AMBRA1	MTCH2	Novel
AMBRA1	PEX11A	Novel
AMBRA1	SLC35C1	Novel
ANXA10	CLCN3	Novel
ANXA11	MPHOSPH9	Novel
ANXA2R	HCN1	Novel

Gene	Interactor
ADAMTSL3	Candidate
AKT1	Candidate
AKT3	Candidate
AMBRA1	Candidate
APOE	Candidate
APOPT1	Candidate
ATP2A2	Candidate
BCL11B	Candidate
BDNF	Candidate
C10orf32	Candidate
C10orf32-ASMT	Candidate
C11orf31	Candidate
C3orf49	Candidate
CACNA1C	Candidate
CACNA1I	Candidate
CACNB2	Candidate
CENPM	Candidate
CHRNA3	Candidate
CHRNA5	Candidate
CHRNA7	Candidate
CLCN3	Candidate
CNNM2	Candidate
CNTN4	Candidate
COMT	Candidate
CSMD1	Candidate
CUL3	Candidate
CYP26B1	Candidate
DAO	Candidate
DAOA	Candidate
DGKI	Candidate
DISC1	Candidate
DPYD	Candidate
DRD2	Candidate
DRD3	Candidate
DRD4	Candidate
DTNBP1	Candidate
EP300-AS1	Candidate
EPC2	Candidate
ETF1	Candidate
FES	Candidate
FLJ45743	Candidate
GALNT10	Candidate
GID4	Candidate
GIGYF2	Candidate

ANXA5	MAD1L1	Novel
APBA3	BDNF	Novel
APC	MAN2A1	Novel
APH1B	NAB2	Novel
APOA5	GRM3	Novel
APOBEC1	CACNA1I	Novel
APOC2	APOE	Novel
APOC4	APOE	Novel
APOE	CD1D	Novel
APOE	CD79A	Novel
APOE	CEACAM1	Novel
APOE	CST1	Novel
APOE	ZBP1	Novel
APOL1	CACNA1I	Novel
APOPT1	IGHV5-78	Novel
APOPT1	INF2	Novel
APOPT1	TDRD9	Novel
APOPT1	TRMT61A	Novel
AQR	CHRNA7	Novel
AREL1	RGS6	Novel
ARF5	MPHOSPH9	Novel
ARF5	MPP6	Novel
ARFGEF2	PTGIS	Novel
ARGLU1	DAOA	Novel
ARHGAP1	BDNF	Novel
ARL4C	GIGYF2	Novel
ARPC3	SRPK2	Novel
AS3MT	CNNM2	Novel
ASB3	VRK2	Novel
ASIC2	SMG6	Novel
ASPH	STAG1	Novel
ATG16L1	GIGYF2	Novel
ATP2A2	FGL2	Novel
ATP2A2	SMCO4	Novel
ATP2A2	UNG	Novel
ATP2B1	DAO	Novel
ATP6V0A2	MPHOSPH9	Novel
BCL11B	BEGAIN	Novel
BCL11B	C14orf132	Novel
BCL11B	WDR25	Novel
BDNF	CD44	Novel
BDNF	CD59	Novel
BDNF	MBTPS2	Novel
BDNF	MPPED2	Novel
BDNF	PSMC3	Novel
BET1	GRM3	Novel
BLM	FES	Novel

GPM6A	Candidate
GRAMD1B	Candidate
GRIN2A	Candidate
GRM3	Candidate
HCN1	Candidate
HTR2A	Candidate
IGSF9B	Candidate
IMMP2L	Candidate
KCNN3	Candidate
KDM3B	Candidate
LINC0147	Candidate
LOC100507431	Candidate
MAD1L1	Candidate
MAN2A1	Candidate
MIR137HG	Candidate
MPHOSPH9	Candidate
MPP6	Candidate
MTHFR	Candidate
NAB2	Candidate
NFATC3	Candidate
NLGN4X	Candidate
NOTCH4	Candidate
NRG1	Candidate
NRGN	Candidate
OTUD7B	Candidate
PAK6	Candidate
PLCH2	Candidate
PPP1R16B	Candidate
PPP3CC	Candidate
PRKD1	Candidate
PRODH	Candidate
PRRG2	Candidate
PTGIS	Candidate
RERE	Candidate
RGS4	Candidate
RGS6	Candidate
SATB2	Candidate
SDCCAG8	Candidate
SHMT2	Candidate
SLC6A3	Candidate
SLC6A4	Candidate
SMG6	Candidate
SNAP91	Candidate
SOX2-OT	Candidate
SRPK2	Candidate
STAG1	Candidate
TCF20	Candidate

BLOC1S3	DTNBP1	Novel
BRE	VRK2	Novel
BRS3	COMT	Novel
BTF3	PTGIS	Novel
BYSL	ETF1	Novel
BYSL	RGS4	Novel
C10orf32	C10orf95	Novel
C10orf32	SFXN2	Novel
C10orf67	CACNB2	Novel
C10orf76	CNNM2	Novel
C11orf31	OOSP2	Novel
C11orf31	PATL1	Novel
C15orf41	PAK6	Novel
C19orf12	ZNF536	Novel
C1orf54	OTUD7B	Novel
C2	TNF	Novel
C22orf29	ZDHHC8	Novel
C22orf46	CENPM	Novel
C4orf27	CLCN3	Novel
C5orf34	HCN1	Novel
CA3	GRM3	Novel
CAB39	GIGYF2	Novel
CABP1	MPHOSPH9	Novel
CACNA1C	CD9	Novel
CACNA1C	RAD51AP1	Novel
CACNA1C	XRCC5	Novel
CACNA1H	PAK6	Novel
CACNA1I	DNAL4	Novel
CACNA1I	GTPBP1	Novel
CACNA1I	HMGXB4	Novel
CACNA1I	JOSD1	Novel
CACNA1I	PICK1	Novel
CACNA1I	PLA2G6	Novel
CACNA1I	SYNGR1	Novel
CACNA1I	TST	Novel
CACNA1I	VPS28	Novel
CACNB2	GPR85	Novel
CACNB2	MSRB2	Novel
CACNB2	NEBL	Novel
CACNB2	NRP1	Novel
CACNB2	PDIA3	Novel
CACNB2	PTER	Novel
CALB2	NFATC3	Novel
CAMTA1	PLCH2	Novel
CAP2	DTNBP1	Novel
CARHSP1	GRIN2A	Novel
CARS	DRD4	Novel

TCF4	Candidate
TMTC1	Candidate
TMX2-CTNND1	Candidate
TNF	Candidate
TSNARE1	Candidate
TYW5	Candidate
VRK2	Candidate
ZDHHC8	Candidate
ZNF536	Candidate
ZNF804A	Candidate
ABI2	Novel
ACACA	Novel
ACOT8	Novel
ACTR1A	Novel
ACVR1B	Novel
ADAM23	Novel
ADAMTS8	Novel
ADCY10	Novel
ADGRA1	Novel
ADGRA2	Novel
AGFG1	Novel
AHNAK2	Novel
AHRR	Novel
AIMP2	Novel
ALDH1B1	Novel
ALDH3A2	Novel
ALDH6A1	Novel
ALG14	Novel
ALPK3	Novel
ALPP	Novel
ALX4	Novel
ANXA10	Novel
ANXA11	Novel
ANXA2R	Novel
ANXA5	Novel
APBA3	Novel
APH1B	Novel
APOA5	Novel
APOBEC1	Novel
APOC1	Novel
APOC2	Novel
APOC4	Novel
APOL1	Novel
AQR	Novel
AREL1	Novel
ARF5	Novel
ARFGEF2	Novel

CASC5	PAK6	Novel
CASP1	DRD2	Novel
CASP3	GPM6A	Novel
CASR	TCF4	Novel
CBWD1	TNF	Novel
CBX5	FES	Novel
CCAR1	DAO	Novel
CCDC134	CENPM	Novel
CCDC91	TMTC1	Novel
CCL16	SMG6	Novel
CCL22	NLGN4X	Novel
CCL4	PRRG2	Novel
CCNA2	DRD2	Novel
CCND2	MAD1L1	Novel
CD3E	DRD2	Novel
CD3G	DRD2	Novel
CDC42SE1	OTUD7B	Novel
CDK2AP1	GRM3	Novel
CDK5RAP1	PPP1R16B	Novel
CDKL3	KDM3B	Novel
CDON	NRGN	Novel
CEBPD	NRG1	Novel
CEBPG	CYP26B1	Novel
CELSR2	NFATC3	Novel
CENPM	SEPT3	Novel
CENPM	DESI1	Novel
CENPM	PHF5A	Novel
CEP104	PLCH2	Novel
CEP170	SDCCAG8	Novel
CEP44	GPM6A	Novel
CEP63	STAG1	Novel
CFTR	SHMT2	Novel
CHCHD3	DGKI	Novel
CHERP	MAD1L1	Novel
CHRNA3	CHRNA5	Novel
CHRNA3	CRABP1	Novel
CHRNA3	ETFA	Novel
CHRNA3	SCAMP2	Novel
CHRNA5	CRABP1	Novel
CHRNA5	LRP2BP	Novel
CHRNA5	SEMA7A	Novel
CHRNA7	MEIS2	Novel
CHST12	MAD1L1	Novel
CLCN3	DECR1	Novel
CLCN3	ETFDH	Novel
CLCN3	FZD3	Novel
CLCN3	GPRC5C	Novel

ARHGAP1	Novel
ARL4C	Novel
ARPC3	Novel
AS3MT	Novel
ASB3	Novel
ASIC2	Novel
ASPH	Novel
ATG16L1	Novel
ATP2B1	Novel
ATP6V0A2	Novel
BEGAIN	Novel
BET1	Novel
BLM	Novel
BLOC1S3	Novel
BNC1	Novel
BRE	Novel
BRS3	Novel
BTF3	Novel
BYSL	Novel
C10orf67	Novel
C10orf76	Novel
C10orf95	Novel
C14orf132	Novel
C15orf41	Novel
C19orf12	Novel
C1orf54	Novel
C2	Novel
C22orf29	Novel
C22orf46	Novel
C4orf27	Novel
C5orf34	Novel
CA3	Novel
CAB39	Novel
CACNA1H	Novel
CALB2	Novel
CAMTA1	Novel
CAP2	Novel
CARS	Novel
CASC5	Novel
CASP1	Novel
CASR	Novel
CBWD1	Novel
CCAR1	Novel
CCDC134	Novel
CCDC91	Novel
CCL16	Novel
CCL22	Novel

CLCN3	GRIA2	Novel
CLCN3	MED23	Novel
CLCN3	NEK1	Novel
CLCN3	PDGFB	Novel
CLCN3	VPS18	Novel
CLDN18	STAG1	Novel
CLUH	SMG6	Novel
CNNM2	DPCD	Novel
CNNM2	FBXL15	Novel
CNNM2	WBP1L	Novel
CNST	DISC1	Novel
CNTN4	CNTN6	Novel
CNTN4	ERAP2	Novel
CNTN4	GHRL	Novel
CNTN4	PRICKLE4	Novel
CNTN4	TBCA	Novel
CNTN5	VRK2	Novel
COMMD6	SHMT2	Novel
COMT	HCK	Novel
COMT	HOPX	Novel
COMT	MRPS5	Novel
COMT	ZNF43	Novel
CPB2	DPYD	Novel
CRABP2	KCNN3	Novel
CSDE1	DPYD	Novel
CSF2	ETF1	Novel
CSGALNACT1	DTNBP1	Novel
CSMD1	NAT1	Novel
CTIF	TCF4	Novel
CTSG	HTR2A	Novel
CTTNBP2	IMMP2L	Novel
CUL3	FAM124B	Novel
CUL3	GYPA	Novel
CUL3	NDUFA10	Novel
CUL3	SRA1	Novel
CUL3	ZAP70	Novel
CXADR	SRPK2	Novel
CYP26B1	GMCL1	Novel
CYP26B1	GTF2H2	Novel
CYP26B1	PCYOX1	Novel
CYP51A1	GRM3	Novel
CYTH2	PRRG2	Novel
DACH1	HTR2A	Novel
DAO	FBXO21	Novel
DAO	PRDM4	Novel
DAO	UNG	Novel
DAOA	MYO16	Novel

CCL4	Novel
CCND2	Novel
CCNT2	Novel
CD1D	Novel
CD3E	Novel
CD3G	Novel
CD44	Novel
CD59	Novel
CD79A	Novel
CD9	Novel
CDC42SE1	Novel
CDK2AP1	Novel
CDK5RAP1	Novel
CDKL3	Novel
CDON	Novel
CEACAM1	Novel
CEBPD	Novel
CEBPG	Novel
CELSR2	Novel
CEP104	Novel
CEP44	Novel
CHCHD3	Novel
CHST12	Novel
CLDN18	Novel
CLUH	Novel
CNST	Novel
CNTN5	Novel
CNTN6	Novel
COMMD6	Novel
CPB2	Novel
CRABP1	Novel
CRABP2	Novel
CREM	Novel
CSDE1	Novel
CSF2	Novel
CSGALNACT1	Novel
CST1	Novel
CTIF	Novel
CTSG	Novel
CTTNBP2	Novel
CXADR	Novel
CYP51A1	Novel
CYTH2	Novel
DACH1	Novel
DDR2	Novel
DECR1	Novel
DEGS1	Novel

DDR2	RGS4	Novel
DDX21	MTHFR	Novel
DEGS1	DISC1	Novel
DEK	DTNBP1	Novel
DGKH	PRKD1	Novel
DGKI	RAB5C	Novel
DHDDS	VRK2	Novel
DIRAS3	HTR2A	Novel
DISC1	EGLN1	Novel
DISC1	EXTL1	Novel
DISC1	TP53BP2	Novel
DKK4	NRG1	Novel
DLL4	PAK6	Novel
DNAJC10	ZNF804A	Novel
DPYD	HPCA	Novel
DPYD	KCNJ4	Novel
DPYD	OVGP1	Novel
DPYD	RPS6KA3	Novel
DPYD	SRC	Novel
DPYD	SULT2A1	Novel
DRD2	E2F6	Novel
DRD2	GPC6	Novel
DRD2	IL10RA	Novel
DRD2	INPPL1	Novel
DRD2	NQO2	Novel
DRD2	PSG7	Novel
DRD2	RBM22	Novel
DRD2	SGSH	Novel
DRD2	VWA5A	Novel
DRD3	GSK3B	Novel
DRD3	POLE2	Novel
DRD3	SPTSSB	Novel
DRD4	ETNK1	Novel
DRD4	KIF2C	Novel
DRD4	MKI67	Novel
DTNBP1	HDGFL1	Novel
DTNBP1	NUP153	Novel
DTNBP1	RNF144B	Novel
DTNBP1	TPMT	Novel
DUSP19	ZNF804A	Novel
EI24	NRGN	Novel
EIF4EBP1	NRG1	Novel
ELAC2	GID4	Novel
EML1	GRIN2A	Novel
ENTPD4	PPP3CC	Novel
EPB41L4A-AS1	MAN2A1	Novel
EPC2	FAP	Novel

DEK	Novel
DESI1	Novel
DGKH	Novel
DHDDS	Novel
DIRAS3	Novel
DKK4	Novel
DNAJC10	Novel
DNAL4	Novel
DPCD	Novel
DUSP19	Novel
E2F6	Novel
EFTUD1	Novel
EGLN1	Novel
EI24	Novel
ELAC2	Novel
EML1	Novel
ENTPD4	Novel
EPB41L4A-AS1	Novel
EPHA2	Novel
ERAP2	Novel
ETFA	Novel
ETFDH	Novel
ETNK1	Novel
EXTL1	Novel
FAM103A1	Novel
FAM106A	Novel
FAM13B	Novel
FAM171B	Novel
FAM53C	Novel
FAM63A	Novel
FAM83D	Novel
FAP	Novel
FBLN1	Novel
FBXL15	Novel
FBXO21	Novel
FBXO42	Novel
FBXO8	Novel
FGFR1	Novel
FGL2	Novel
FHL2	Novel
FSCN1	Novel
FURIN	Novel
FZD3	Novel
GABARAPL2	Novel
GEMIN5	Novel
GFRA2	Novel
GGT3P	Novel

EPC2	MMADHC	Novel
EPC2	NMI	Novel
EPHA2	GIGYF2	Novel
ETF1	MYOZ2	Novel
ETF1	UBE4B	Novel
ETF1	WFDC1	Novel
FAM106A	GID4	Novel
FAM13B	KDM3B	Novel
FAM171B	ZNF804A	Novel
FAM53C	KDM3B	Novel
FAM63A	OTUD7B	Novel
FAM83D	PPP1R16B	Novel
FBLN1	TCF20	Novel
FBXO42	RERE	Novel
FBXO8	GPM6A	Novel
FES	FURIN	Novel
FES	MAN2A2	Novel
FES	NLGN4X	Novel
FES	PEX11A	Novel
FES	STARD5	Novel
FES	VANGL1	Novel
FES	ZNF117	Novel
FGFR1	NRG1	Novel
FHL2	MTHFR	Novel
FSCN1	MAD1L1	Novel
GABARAPL2	GPM6A	Novel
GALNT10	GEMIN5	Novel
GALNT10	LCP2	Novel
GALNT10	SAP30L	Novel
GALNT10	SPINK7	Novel
GFRA2	PPP3CC	Novel
GGT3P	PRODH	Novel
GID4	LRRC48	Novel
GIGYF2	METTL21A	Novel
GIGYF2	NMUR1	Novel
GJD2	PAK6	Novel
GMFG	PTGIS	Novel
GNAS	PTGIS	Novel
GPR137B	NLGN4X	Novel
GPR88	MIR137HG	Novel
GRIN2A	ID4	Novel
GRIN2A	PCDH8	Novel
GRIN2A	PLA2G10	Novel
GRIN2A	PRM1	Novel
GRIN2A	RNF40	Novel
GRM3	OCM2	Novel
GRM3	SLC22A1	Novel

GHRL	Novel
GJD2	Novel
GMFG	Novel
GNAS	Novel
GOLGA2P10	Novel
GPC6	Novel
GPR137B	Novel
GPR85	Novel
GPR88	Novel
GPRC5C	Novel
GSC2	Novel
GTF2H2	Novel
GTPBP1	Novel
GUK1	Novel
GYPA	Novel
HCAR3	Novel
HCK	Novel
HDGFL1	Novel
HDGFRP3	Novel
HMGXB4	Novel
HOPX	Novel
HOXA10	Novel
HPCA	Novel
HRH1	Novel
HS3ST1	Novel
HSD17B12	Novel
IDH3A	Novel
IGHV5-78	Novel
IL10RA	Novel
ILF2	Novel
INF2	Novel
INPPL1	Novel
ISG20	Novel
ITM2C	Novel
JOSD1	Novel
KBTBD4	Novel
KCNC4	Novel
KCNJ3	Novel
KCNJ4	Novel
KCNK4	Novel
KIAA1715	Novel
KIF2C	Novel
KMT2D	Novel
KNTC1	Novel
KXD1	Novel
LAMP3	Novel
LCP1	Novel

GRM3	ZNF219	Novel
GRM3	ZNF804B	Novel
GSC2	PRODH	Novel
GSK3A	MPHOSPH9	Novel
GUK1	STAG1	Novel
HAX1	KCNN3	Novel
HCAR3	MPHOSPH9	Novel
HCN1	LOC153684	Novel
HOXA10	MAD1L1	Novel
HOXA10	NAB2	Novel
HRH1	NAB2	Novel
HS3ST1	PRKD1	Novel
HSPB7	RERE	Novel
HTR2A	PCDH9	Novel
HTR2A	RFXAP	Novel
HTR2A	SUGT1	Novel
IGSF9B	NCAPD3	Novel
ILF2	KCNN3	Novel
INPP5K	SMG6	Novel
ISG20	NRG1	Novel
ITM2C	PRODH	Novel
KCNC4	TNF	Novel
KCNJ3	NFATC3	Novel
KCNK4	NRG1	Novel
KCNN3	SLC27A3	Novel
KCNN3	UBAP2L	Novel
KDM3B	PCDH12	Novel
KDM3B	PCDHGB7	Novel
KDM3B	PCDHGC4	Novel
KDM3B	PCDHGC5	Novel
KDM3B	REEP2	Novel
KIAA1715	RERE	Novel
KMT2D	SHMT2	Novel
KNTC1	MPHOSPH9	Novel
KXD1	SRPK2	Novel
LAMP3	SOX2-OT	Novel
LCP1	NLGN4X	Novel
LEPROTL1	PPP3CC	Novel
LIPF	NRG1	Novel
LIPG	TCF4	Novel
LPPR5	MIR137HG	Novel
LRP1	NAB2	Novel
LTB4R2	PTGIS	Novel
MAD1L1	NXPH1	Novel
MAD1L1	PIN4	Novel
MAD1L1	PRKAR1B	Novel
MAD1L1	RPA3	Novel

LCP2	Novel
LEPROTL1	Novel
LIPF	Novel
LIPG	Novel
LOC153684	Novel
LPPR5	Novel
LRP2BP	Novel
LRRC48	Novel
LTB4R2	Novel
MAN2A2	Novel
MARS2	Novel
MBTPS2	Novel
MCEE	Novel
MED23	Novel
MEIS2	Novel
METTL21A	Novel
MICAL3	Novel
MIS12	Novel
MITF	Novel
MKI67	Novel
MKRN2	Novel
MLEC	Novel
MMADHC	Novel
MMP23B	Novel
MOB1B	Novel
MPPED2	Novel
MPZ	Novel
MRPL12	Novel
MRPL19	Novel
MRPL36	Novel
MRPL40	Novel
MRPS5	Novel
MSH5	Novel
MSL2	Novel
MSRB2	Novel
MTCH2	Novel
MYL9	Novel
MYO16	Novel
MYOCD	Novel
MYOZ2	Novel
NAT1	Novel
NCAPD3	Novel
NDUFA10	Novel
NEK1	Novel
NEU1	Novel
NFKBIL1	Novel
NFX1	Novel

MAD1L1	S100P	Novel
MAD1L1	STAM	Novel
MAD1L1	TTYH3	Novel
MAD1L1	TULP4	Novel
MARS2	TYW5	Novel
MCEE	PPP1R16B	Novel
MICAL3	ZDHHC8	Novel
MIR137HG	SLC44A3	Novel
MIR137HG	TMEM56	Novel
MITF	RERE	Novel
MKRN2	MPP6	Novel
MLEC	MPHOSPH9	Novel
MMP23B	MTHFR	Novel
MOB1B	TNF	Novel
MPHOSPH9	MRPL19	Novel
MPHOSPH9	RAB35	Novel
MPHOSPH9	SNRNP35	Novel
MPHOSPH9	SRSF9	Novel
MPHOSPH9	TRA2A	Novel
MPP6	OSBPL3	Novel
MPP6	PTF1A	Novel
MPP6	TACR3	Novel
MPZ	RGS4	Novel
MRPL12	SMG6	Novel
MRPL36	SLC6A3	Novel
MRPL40	ZDHHC8	Novel
MSH5	NOTCH4	Novel
MSL2	STAG1	Novel
MTHFR	PRKCZ	Novel
MTHFR	TP73	Novel
MTHFR	ZBTB17	Novel
MTHFR	ZNF239	Novel
MYL9	PPP1R16B	Novel
MYO1A	NAB2	Novel
MYOCD	NFATC3	Novel
NAB2	RDH5	Novel
NAB2	SCN1A	Novel
NEU1	NOTCH4	Novel
NFATC3	SEC24A	Novel
NFATC3	SNX20	Novel
NFATC3	TOR1B	Novel
NFKBIL1	NOTCH4	Novel
NFX1	PPP1R16B	Novel
NFYA	NOTCH4	Novel
NKD2	SHMT2	Novel
NKG7	PRRG2	Novel
NLGN4X	ZSCAN31	Novel

NFYA	Novel
NKD2	Novel
NKG7	Novel
NMI	Novel
NMUR1	Novel
NQO2	Novel
NRP1	Novel
NUP153	Novel
NUP35	Novel
NXPH1	Novel
OCM2	Novel
OOSP2	Novel
OR10J1	Novel
OSBPL3	Novel
OSMR	Novel
OVCH1	Novel
OVGP1	Novel
PAF1	Novel
PATL1	Novel
PBX2	Novel
PCDH12	Novel
PCDH8	Novel
PCDH9	Novel
PCDHGB7	Novel
PCDHGC4	Novel
PCDHGC5	Novel
PCYOX1	Novel
PDGFB	Novel
PDIA3	Novel
PEX10	Novel
PEX11A	Novel
PFDN2	Novel
PGM3	Novel
PHF5A	Novel
PILRB	Novel
PIN4	Novel
PKHD1	Novel
PLA2G10	Novel
PLA2G6	Novel
PLD3	Novel
PLEKHB2	Novel
POLE2	Novel
POLR2I	Novel
PPIL2	Novel
PPP1R11	Novel
PRDM4	Novel
PRICKLE4	Novel

NOTCH4	PBX2	Novel
NOTCH4	PSMB8	Novel
NOTCH4	SLC27A4	Novel
NOTCH4	TDP2	Novel
NOTCH4	WWP2	Novel
NRGN	PLEKHB2	Novel
NUP35	ZNF804A	Novel
OR10J1	SRPK2	Novel
OSMR	RGS4	Novel
OTUD7B	TNFAIP8L2	Novel
OVCH1	TMTC1	Novel
PAF1	RERE	Novel
PAK6	SP3	Novel
PAK6	SPG11	Novel
PAK6	SUMO2	Novel
PEX10	PLCH2	Novel
PFDN2	RGS4	Novel
PGM3	SNAP91	Novel
PIAS2	TCF4	Novel
PILRB	TNF	Novel
PKHD1	PTGIS	Novel
PLCH2	TP73-AS1	Novel
PLD3	VRK2	Novel
POLR2I	ZNF536	Novel
PPIL2	ZDHHC8	Novel
PPP1R11	TNF	Novel
PPP1R16B	SSR4	Novel
PPP1R16B	TTI1	Novel
PPP1R16B	TUFM	Novel
PPP2R1A	PRRG2	Novel
PPP3CC	SLC18A1	Novel
PPP3CC	SORBS3	Novel
PRKACA	TCF4	Novel
PRKAG1	SHMT2	Novel
PRKD1	RGS1	Novel
PRKD1	YY1	Novel
PRO2964	ZNF804A	Novel
PRODH	SEPT5	Novel
PRODH	RANBP1	Novel
PRODH	SKIV2L	Novel
PRODH	TBX1	Novel
PRPF8	SMG6	Novel
PRRG2	RTN4RL1	Novel
PRRG2	SGTA	Novel
PRRG2	SNX15	Novel
PTGIS	TMSB4XP6	Novel
PTTG1	VRK2	Novel

PRKAG1	Novel
PRKAR1B	Novel
PRM1	Novel
PRO2964	Novel
PRPF8	Novel
PSG7	Novel
PSMB8	Novel
PSMC3	Novel
PTER	Novel
PTTG1	Novel
RAB35	Novel
RAB5C	Novel
RAD51AP1	Novel
RANBP1	Novel
RAP1GAP2	Novel
RBM22	Novel
RDH5	Novel
REEP2	Novel
RFXAP	Novel
RGS1	Novel
RGS7	Novel
RIPPLY2	Novel
RND1	Novel
RNF144B	Novel
RNF40	Novel
RPA3	Novel
RPL18A	Novel
RRM1	Novel
RTN4R	Novel
RTN4RL1	Novel
RWDD2A	Novel
S100P	Novel
SAP30L	Novel
SATB2-AS1	Novel
SCAF11	Novel
SCAMP2	Novel
SCN1A	Novel
SDHC	Novel
SEC24A	Novel
SEMA7A	Novel
SEPT3	Novel
SEPT5	Novel
SETBP1	Novel
SFXN2	Novel
SGSH	Novel
SGTA	Novel
SKIV2L	Novel

RAP1GAP2	SMG6	Novel
RERE	ZBTB17	Novel
RERE	ZNF239	Novel
RGS4	RRM1	Novel
RGS4	SDHC	Novel
RGS4	UAP1	Novel
RGS6	TMED10	Novel
RGS7	SDCCAG8	Novel
RIPPLY2	SNAP91	Novel
RND1	SHMT2	Novel
RPL18A	TCF4	Novel
RTN4R	ZDHHC8	Novel
RWDD2A	SNAP91	Novel
SATB2-AS1	TYW5	Novel
SCAF11	SHMT2	Novel
SETBP1	TCF4	Novel
SHMT2	TUBA1C	Novel
SLC6A4	SMAD2	Novel
SMAD2	TCF4	Novel
SMC1B	STAG1	Novel
SMG6	SNAI2	Novel
SRPK2	TRIM4	Novel
ST5	TCF4	Novel
STAG1	VDR	Novel
TAPBP	TNF	Novel
TBC1D22A	TCF20	Novel
TCF20	TRAM1	Novel
TCF20	TST	Novel
TCF20	UGT2B4	Novel
TCF20	ZMYND11	Novel
TNF	TNXB	Novel
TOP1MT	TSNARE1	Novel
TSHZ3	ZNF536	Novel
TSNARE1	ZNF707	Novel
UBE2E3	ZNF804A	Novel
ZC3H15	ZNF804A	Novel
ZNF804A	ZSWIM2	Novel
A2M	APOE	Known
AATF	MPP6	Known
ABCA1	MPP6	Known
ABI3	DTNBP1	Known
ABL1	PRKD1	Known
ABL1	SRPK2	Known
ACTG1	APOE	Known
ACTG1	DISC1	Known
ACTN1	GRIN2A	Known
ACTN2	DISC1	Known

SLC18A1	Novel
SLC22A1	Novel
SLC27A3	Novel
SLC27A4	Novel
SLC35C1	Novel
SLC44A3	Novel
SMC1B	Novel
SMCO4	Novel
SNAI2	Novel
SNX15	Novel
SNX20	Novel
SP3	Novel
SPG11	Novel
SPINK7	Novel
SPTSSB	Novel
SRA1	Novel
SSR4	Novel
ST5	Novel
STAM	Novel
STARD5	Novel
SUGT1	Novel
SULT2A1	Novel
SYNGR1	Novel
TACR3	Novel
TAPBP	Novel
TBC1D22A	Novel
TBCA	Novel
TBX1	Novel
TDRD9	Novel
TMED10	Novel
TMEM56	Novel
TMSB4XP6	Novel
TNFAIP8L2	Novel
TNXB	Novel
TOP1MT	Novel
TOR1B	Novel
TP53BP2	Novel
TP73	Novel
TP73-AS1	Novel
TPMT	Novel
TRAM1	Novel
TRIM4	Novel
TRMT61A	Novel
TST	Novel
TTI1	Novel
TTYH3	Novel
TUBA1C	Novel

ADAM17	TNF	Known
ADAMTSL3	KRT40	Known
ADAMTSL3	KRTAP10-3	Known
ADAMTSL3	KRTAP10-8	Known
ADAMTSL3	KRTAP2-4	Known
ADAMTSL3	NOTCH2NL	Known
ADCY6	CHRNA7	Known
ADORA2A	DRD2	Known
ADPRH	SRPK2	Known
AES	NAB2	Known
AES	SRPK2	Known
AES	TCF4	Known
AGTPBP1	DISC1	Known
AKAP6	DISC1	Known
AKAP6	DTNBP1	Known
AKAP9	DISC1	Known
AKT1	ACAP1	Known
AKT1	AHNAK	Known
AKT1	AKT1S1	Known
AKT1	AKT2	Known
AKT1	AKTIP	Known
AKT1	ALYREF	Known
AKT1	APLP2	Known
AKT1	APOH	Known
AKT1	APP	Known
AKT1	APPL1	Known
AKT1	AR	Known
AKT1	ARFIP2	Known
AKT1	ARHGAP29	Known
AKT1	ARHGAP32	Known
AKT1	ATXN1	Known
AKT1	BAD	Known
AKT1	BCL10	Known
AKT1	BCL2L1	Known
AKT1	BCL2L11	Known
AKT1	BPGM	Known
AKT1	BRAF	Known
AKT1	BRCA1	Known
AKT1	CAMKK1	Known
AKT1	CARHSP1	Known
AKT1	CASP3	Known
AKT1	CASP9	Known
AKT1	CCDC88A	Known
AKT1	CDKN1A	Known
AKT1	CDKN1B	Known
AKT1	CDKN1C	Known
AKT1	CHEK1	Known

TUFM	Novel
TULP4	Novel
UAP1	Novel
UBAP2L	Novel
UBE4B	Novel
UGT2B4	Novel
UNG	Novel
VANGL1	Novel
VDR	Novel
VPS18	Novel
VWA5A	Novel
WBP1L	Novel
WDR25	Novel
WFDC1	Novel
WWP2	Novel
YY1	Novel
ZAP70	Novel
ZBP1	Novel
ZBTB17	Novel
ZC3H15	Novel
ZMYND11	Novel
ZNF117	Novel
ZNF219	Novel
ZNF239	Novel
ZNF43	Novel
ZNF707	Novel
ZNF804B	Novel
ZSCAN31	Novel
ZSWIM2	Novel
A2M	Known
AATF	Known
ABCA1	Known
ABI3	Known
ABL1	Known
ABTB1	Known
ABTB2	Known
ACAP1	Known
ACE	Known
ACIN1	Known
ACTG1	Known
ACTN1	Known
ACTN2	Known
ADAM17	Known
ADAM19	Known
ADAM9	Known
ADAP1	Known
ADCY6	Known

AKT1	CHN2	Known
AKT1	CHUK	Known
AKT1	CLIP3	Known
AKT1	CLK2	Known
AKT1	CREB1	Known
AKT1	CREBBP	Known
AKT1	CSNK2A1	Known
AKT1	CTNNB1	Known
AKT1	CYLD	Known
AKT1	DAB2IP	Known
AKT1	DCTN1	Known
AKT1	DLC1	Known
AKT1	DNAJB1	Known
AKT1	DNMT1	Known
AKT1	EEF1G	Known
AKT1	EGFR	Known
AKT1	EIF4EBP1	Known
AKT1	EP300	Known
AKT1	ESR1	Known
AKT1	ESR2	Known
AKT1	EZH2	Known
AKT1	FANCA	Known
AKT1	FOXO1	Known
AKT1	FOXO3	Known
AKT1	FOXO4	Known
AKT1	GAB2	Known
AKT1	GATA1	Known
AKT1	GATA2	Known
AKT1	GRB10	Known
AKT1	GSK3A	Known
AKT1	GSK3B	Known
AKT1	HIST2H2BE	Known
AKT1	HMOX1	Known
AKT1	HSP90AA1	Known
AKT1	HSP90AB1	Known
AKT1	HSPB1	Known
AKT1	HTT	Known
AKT1	IKBKB	Known
AKT1	ILK	Known
AKT1	IMPDH2	Known
AKT1	IRAK1	Known
AKT1	IRS1	Known
AKT1	ITGB3	Known
AKT1	ITPR1	Known
AKT1	ITPR3	Known
AKT1	JADE1	Known
AKT1	KAT2B	Known

ADGRG5	Known
ADORA2A	Known
ADPRH	Known
AES	Known
AGO3	Known
AGTPBP1	Known
AHCYL1	Known
AHNAK	Known
AKAP13	Known
AKAP6	Known
AKAP9	Known
AKT1S1	Known
AKT2	Known
AKTIP	Known
ALB	Known
ALG13	Known
ALKBH3	Known
ALOX5	Known
ALYREF	Known
AMMECR1L	Known
AMOTL2	Known
AMPH	Known
ANKH	Known
ANKS1B	Known
AOC1	Known
AP1M1	Known
AP2A2	Known
AP2B1	Known
AP4M1	Known
APC	Known
APEX1	Known
APLP2	Known
APOBEC3C	Known
APOH	Known
APP	Known
APPL1	Known
AQP1	Known
AR	Known
ARFGAP1	Known
ARFIP2	Known
ARGLU1	Known
ARHGAP12	Known
ARHGAP18	Known
ARHGAP29	Known
ARHGAP32	Known
ARHGEF1	Known
ARHGEF12	Known

AKT1	KAT6A	Known
AKT1	KRT10	Known
AKT1	KTN1	Known
AKT1	LRRK2	Known
AKT1	MAP2K4	Known
AKT1	MAP3K11	Known
AKT1	MAP3K5	Known
AKT1	MAP3K8	Known
AKT1	MAPK14	Known
AKT1	MAPK8IP1	Known
AKT1	MAPKAPK2	Known
AKT1	MAPT	Known
AKT1	MDM2	Known
AKT1	MDM4	Known
AKT1	METTL1	Known
AKT1	MS4A2	Known
AKT1	MST1R	Known
AKT1	MTCP1	Known
AKT1	MTOR	Known
AKT1	MUL1	Known
AKT1	MXD1	Known
AKT1	NCOA4	Known
AKT1	NCOR2	Known
AKT1	NF2	Known
AKT1	NOS3	Known
AKT1	NOTCH1	Known
AKT1	NR4A1	Known
AKT1	PAK1	Known
AKT1	PAK6	Known
AKT1	PDE3B	Known
AKT1	PDK2	Known
AKT1	PDPK1	Known
AKT1	PEA15	Known
AKT1	PFKFB1	Known
AKT1	PFKFB2	Known
AKT1	PHB2	Known
AKT1	PI4K2B	Known
AKT1	PIAS1	Known
AKT1	PIAS2	Known
AKT1	PIK3R1	Known
AKT1	PKN2	Known
AKT1	PLCG1	Known
AKT1	PLEKHO1	Known
AKT1	PLXNA1	Known
AKT1	PPARGC1B	Known
AKT1	PPL	Known
AKT1	PPM1A	Known

ARIH2	Known
ARL6IP1	Known
ARL6IP4	Known
ARMC7	Known
ARPC1B	Known
ASCL1	Known
ASCL3	Known
ASCL4	Known
ASPSCR1	Known
ATAT1	Known
ATF4	Known
ATF5	Known
ATF7IP	Known
ATN1	Known
ATP6V1B2	Known
ATXN1	Known
AURKA	Known
BAD	Known
BAG5	Known
BAIAP2	Known
BARD1	Known
BCAR1	Known
BCAS2	Known
BCL10	Known
BCL2	Known
BCL2L1	Known
BCL2L11	Known
BCL2L2	Known
BCR	Known
BECN1	Known
BGN	Known
BICD1	Known
BLOC1S5	Known
BLOC1S6	Known
BMPR1B	Known
BPGM	Known
BRAF	Known
BRCA1	Known
BRD3	Known
BRF1	Known
BRK1	Known
BTBD10	Known
BTK	Known
BZRAP1	Known
C14orf166	Known
C16orf78	Known
C17orf85	Known

AKT1	PPP1CA	Known
AKT1	PPP2CA	Known
AKT1	PPP2R1A	Known
AKT1	PPP2R4	Known
AKT1	PRG2	Known
AKT1	PRKCQ	Known
AKT1	PRKCZ	Known
AKT1	PRKDC	Known
AKT1	PTEN	Known
AKT1	PTPN1	Known
AKT1	RAB3D	Known
AKT1	RAC1	Known
AKT1	RAF1	Known
AKT1	RARA	Known
AKT1	RGCC	Known
AKT1	RHEBL1	Known
AKT1	RICTOR	Known
AKT1	RNF11	Known
AKT1	RNF115	Known
AKT1	RPS6KB1	Known
AKT1	RQCD1	Known
AKT1	S1PR1	Known
AKT1	SETDB1	Known
AKT1	SH2B2	Known
AKT1	SH3RF1	Known
AKT1	SIRT1	Known
AKT1	SIRT6	Known
AKT1	SKI	Known
AKT1	SKP2	Known
AKT1	SMAD2	Known
AKT1	SMAD3	Known
AKT1	SMAD4	Known
AKT1	SMAD7	Known
AKT1	SMARCB1	Known
AKT1	SMARCC1	Known
AKT1	SNCA	Known
AKT1	SORBS2	Known
AKT1	SORBS3	Known
AKT1	SRC	Known
AKT1	STAT1	Known
AKT1	STK3	Known
AKT1	STK4	Known
AKT1	TBC1D4	Known
AKT1	TCL1A	Known
AKT1	TCL1B	Known
AKT1	TCL6	Known
AKT1	TERF2IP	Known

C18orf25	Known
C19orf52	Known
C19orf66	Known
C1QBP	Known
C1orf109	Known
C1orf35	Known
C4orf46	Known
C9orf171	Known
CABIN1	Known
CABP1	Known
CABP5	Known
CACNB3	Known
CACNG2	Known
CADPS	Known
CADPS2	Known
CALM1	Known
CALM2	Known
CALM3	Known
CALR	Known
CAMK1D	Known
CAMK2A	Known
CAMK2B	Known
CAMKK1	Known
CAND1	Known
CANX	Known
CARHSP1	Known
CASK	Known
CASP3	Known
CASP9	Known
CBFA2T2	Known
CBLN4	Known
CBX5	Known
CCDC101	Known
CCDC136	Known
CCDC141	Known
CCDC146	Known
CCDC153	Known
CCDC24	Known
CCDC28A	Known
CCDC33	Known
CCDC53	Known
CCDC88A	Known
CCDC94	Known
CCDC97	Known
CCHCR1	Known
CCM2	Known
CCNA2	Known

AKT1	TERT	Known
AKT1	THEM4	Known
AKT1	TNFSF11	Known
AKT1	TOPBP1	Known
AKT1	TRIB3	Known
AKT1	TRIM13	Known
AKT1	TSC1	Known
AKT1	TSC2	Known
AKT1	TTC3	Known
AKT1	USP4	Known
AKT1	UXS1	Known
AKT1	VEGFA	Known
AKT1	VIM	Known
AKT1	WNK1	Known
AKT1	WNK4	Known
AKT1	XIAP	Known
AKT1	YAP1	Known
AKT1	YBX1	Known
AKT1	YWHAZ	Known
AKT1	ZFP36L1	Known
AKT1	ZHX1	Known
AKT3	NEDD4L	Known
ALB	APOE	Known
ALOX5	MAD1L1	Known
AMPH	PPP3CC	Known
AOC1	DAO	Known
AP2A2	SNAP91	Known
AP2B1	SNAP91	Known
APC	GIGYF2	Known
APEX1	SRPK2	Known
APOE	ANKH	Known
APOE	APP	Known
APOE	ARFGAP1	Known
APOE	C19orf52	Known
APOE	CDC37	Known
APOE	CNTF	Known
APOE	CSNK2A1	Known
APOE	CTSB	Known
APOE	CYP2C18	Known
APOE	CYP2C8	Known
APOE	ECSIT	Known
APOE	ELAVL1	Known
APOE	EPN2	Known
APOE	FARSA	Known
APOE	FBXL12	Known
APOE	FOXG1	Known
APOE	FXYD7	Known

CCND1	Known
CCND3	Known
CCNE1	Known
CCT2	Known
CCT4	Known
CDC25A	Known
CDC27	Known
CDC34	Known
CDC37	Known
CDC42	Known
CDC42EP4	Known
CDC5L	Known
CDC73	Known
CDCA7L	Known
CDK1	Known
CDK5	Known
CDK5RAP3	Known
CDK6	Known
CDK7	Known
CDKN1A	Known
CDKN1B	Known
CDKN1C	Known
CDKN2C	Known
CEP126	Known
CEP170	Known
CEP55	Known
CEP57L1	Known
CEP63	Known
CEP70	Known
CFTR	Known
CHAF1A	Known
CHCHD2	Known
CHD2	Known
CHEK1	Known
CHERP	Known
CHIC2	Known
CHN2	Known
CHRNB4	Known
CHTOP	Known
CHUK	Known
CIT	Known
CKS1B	Known
CLDN19	Known
CLIC6	Known
CLIP3	Known
CLK1	Known
CLK2	Known

APOE	GCDH	Known
APOE	HTRA1	Known
APOE	IFIT3	Known
APOE	IFIT5	Known
APOE	IQSEC1	Known
APOE	LCAT	Known
APOE	LDLR	Known
APOE	LONP1	Known
APOE	LOXL4	Known
APOE	LRP1	Known
APOE	LRP2	Known
APOE	LRP8	Known
APOE	MAP2	Known
APOE	MAPT	Known
APOE	MAST1	Known
APOE	MID1IP1	Known
APOE	NEFM	Known
APOE	NOS3	Known
APOE	PCMT1	Known
APOE	PDCD4	Known
APOE	PLEKHA6	Known
APOE	PLTP	Known
APOE	PRAM1	Known
APOE	PRDX2	Known
APOE	PRNP	Known
APOE	PSEN1	Known
APOE	RHEB	Known
APOE	RNF32	Known
APOE	RPL4	Known
APOE	SCARB1	Known
APOE	ST13	Known
APOE	TYRO3	Known
APOE	VLDLR	Known
APOE	ZNF558	Known
APP	BDNF	Known
APP	CHRNA7	Known
APP	CUL3	Known
APP	KCNN3	Known
APP	PAK6	Known
APP	PPP3CC	Known
APP	PTGIS	Known
APP	SHMT2	Known
APP	SRPK2	Known
AQP1	TCF4	Known
AR	PAK6	Known
AR	TCF20	Known
AR	TCF4	Known

CLK3	Known
CLU	Known
CMTM5	Known
CMYA5	Known
CNTF	Known
COIL	Known
COL4A1	Known
COMMD1	Known
COPB1	Known
COPB2	Known
COPS2	Known
COPS5	Known
COPS7A	Known
CPE	Known
CREB1	Known
CREBBP	Known
CRX	Known
CSAG1	Known
CSF1	Known
CSF2RB	Known
CSNK1A1	Known
CSNK2A1	Known
CSNK2B	Known
CTBP1	Known
CTNNB1	Known
CTNNBL1	Known
CTSB	Known
CWF19L2	Known
CYLD	Known
CYP2C18	Known
CYP2C8	Known
DAB2IP	Known
DAW1	Known
DBF4B	Known
DCN	Known
DCTN1	Known
DCTN2	Known
DCUN1D1	Known
DCUN1D2	Known
DCUN1D3	Known
DCUN1D4	Known
DCUN1D5	Known
DDX21	Known
DDX46	Known
DDX6	Known
DEF6	Known
DGCR6	Known

ARFIP2	DTNBP1	Known
ARIH2	DISC1	Known
ASCL1	TCF4	Known
ATF4	DISC1	Known
ATF5	DISC1	Known
ATP2A2	BCL2	Known
ATP2A2	CAMK2A	Known
ATP2A2	HGS	Known
ATP2A2	IRS1	Known
ATP2A2	IRS2	Known
ATP2A2	MDM2	Known
ATP2A2	PLN	Known
ATP2A2	S100A1	Known
ATP2A2	SPP1	Known
ATP6V1B2	TCF4	Known
ATXN1	ZNF804A	Known
BARD1	CNTN4	Known
BCL2	PPP3CC	Known
BCL2L2	TCF4	Known
BCR	FES	Known
BDNF	AGO3	Known
BDNF	CADPS2	Known
BDNF	CPE	Known
BDNF	ESR1	Known
BDNF	F11R	Known
BDNF	INPP5K	Known
BDNF	JUNB	Known
BDNF	MBTPS1	Known
BDNF	NCAM1	Known
BDNF	NOS3	Known
BDNF	NTF3	Known
BDNF	NTRK2	Known
BDNF	SORT1	Known
BECN1	AMBRA1	Known
BGN	TNF	Known
BICD1	DISC1	Known
BLOC1S5	DTNBP1	Known
BLOC1S6	DTNBP1	Known
BMPR1B	PPP3CC	Known
BRCA1	CNTN4	Known
BRF1	DTNBP1	Known
BRK1	DTNBP1	Known
BTK	PRKD1	Known
BZRAP1	DISC1	Known
C19orf66	DTNBP1	Known
C1QBP	PRKD1	Known
CACNA1C	CABP1	Known

DGCR6L	Known
DHX8	Known
DIS3	Known
DLC1	Known
DLG1	Known
DLG2	Known
DLG3	Known
DLG4	Known
DLGAP1	Known
DLGAP2	Known
DLL4	Known
DMAP1	Known
DMD	Known
DMRT3	Known
DNAJB1	Known
DNAJC7	Known
DNAJC8	Known
DNM2	Known
DNMT1	Known
DOK1	Known
DPYSL2	Known
DPYSL3	Known
DPYSL5	Known
DRAP1	Known
DRG1	Known
DST	Known
DTNA	Known
DTNB	Known
DYNC1H1	Known
DYNLL1	Known
DYNLRB1	Known
DYRK4	Known
ECM1	Known
ECSIT	Known
EED	Known
EEF1B2	Known
EEF1G	Known
EEF2	Known
EFEMP1	Known
EFEMP2	Known
EFHC1	Known
EFNB1	Known
EGF	Known
EGFL7	Known
EGFR	Known
EGLN2	Known
EGR1	Known

CACNA1C	CACNB3	Known
CACNA1C	COPS5	Known
CACNA1C	GNB1	Known
CACNA1C	HDAC4	Known
CACNA1C	PCBD1	Known
CACNA1C	PPM1A	Known
CACNA1C	PRKACA	Known
CACNA1C	RIMS1	Known
CACNA1C	RYR2	Known
CACNA1C	SRI	Known
CACNB2	PRKACA	Known
CACNB2	REM1	Known
CALM1	DRD2	Known
CALM1	GRM3	Known
CALM1	KCNN3	Known
CALM1	NRGN	Known
CALM1	RGS4	Known
CALM1	STAG1	Known
CALM1	TCF4	Known
CALM2	TCF4	Known
CALM3	TCF4	Known
CALR	SLC6A4	Known
CAMK2A	GRIN2A	Known
CAMK2B	GRIN2A	Known
CANX	SLC6A4	Known
CBX5	BCL11B	Known
CCDC146	DTNBP1	Known
CCDC53	DTNBP1	Known
CCHCR1	DTNBP1	Known
CCNA2	DTNBP1	Known
CCND1	CUL3	Known
CCND3	DTNBP1	Known
CCNE1	CUL3	Known
CDC25A	CUL3	Known
CDC27	COMT	Known
CDC34	CUL3	Known
CDC42	PAK6	Known
CDC42	VRK2	Known
CDC5L	DISC1	Known
CDK1	PAK6	Known
CDK5	GRIN2A	Known
CDK6	NFATC3	Known
CDK7	SRPK2	Known
CDKN1A	TCF4	Known
CDKN2C	TCF4	Known
CEP170	DISC1	Known
CFTR	CLCN3	Known

EGR2	Known
EHMT2	Known
EIF1AY	Known
EIF3F	Known
EIF3G	Known
EIF3H	Known
EIF4E2	Known
EIF4EBP1	Known
EIF5	Known
ELAVL1	Known
ELOVL7	Known
ENOX2	Known
EP300	Known
EPAS1	Known
EPB41	Known
EPB41L1	Known
EPB41L2	Known
EPB41L3	Known
EPB41L4A	Known
EPHB6	Known
EPN2	Known
EPS15	Known
EPS8L3	Known
ERBB2	Known
ERBB3	Known
ERBB4	Known
ERCC3	Known
ERN1	Known
ESR1	Known
ESR2	Known
EVI5L	Known
EXOC1	Known
EXOC4	Known
EXOC7	Known
EXOSC1	Known
EXOSC10	Known
EXOSC2	Known
EXOSC4	Known
EXOSC5	Known
EXOSC6	Known
EXOSC7	Known
EXOSC8	Known
EXOSC9	Known
EZH2	Known
F11R	Known
FAH	Known
FAM124B	Known

CHD2	SRPK2	Known
CHRNA3	CHRNB4	Known
CHRNA3	UBQLN1	Known
CHRNA5	CHRNB4	Known
CHRNA7	ATXN1	Known
CHRNA7	PIK3R1	Known
CHUK	SRPK2	Known
CIT	DISC1	Known
CKS1B	TCF4	Known
CLCN3	GOPC	Known
CLCN3	PDZK1	Known
CLCN3	SLC9A3R1	Known
CLK1	SRPK2	Known
CLK2	SRPK2	Known
CLK3	SRPK2	Known
CLU	DISC1	Known
COIL	MAD1L1	Known
COL4A1	DISC1	Known
COMT	ACE	Known
COMT	KRT31	Known
COMT	KRT40	Known
COMT	KRTAP5-9	Known
COMT	LITAF	Known
COMT	RGS2	Known
COMT	TRIP13	Known
COMT	VKORC1	Known
COMT	XRN2	Known
COPB1	RGS4	Known
CREBBP	KDM3B	Known
CRX	PPP1R16B	Known
CSF1	TNF	Known
CSF2RB	FES	Known
CSNK1A1	NFATC3	Known
CSNK2B	PPP3CC	Known
CSNK2B	SHMT2	Known
CTBP1	TCF4	Known
CTNNB1	TCF4	Known
CUL3	ABTB1	Known
CUL3	ABTB2	Known
CUL3	ARHGEF12	Known
CUL3	BTBD10	Known
CUL3	CAMK1D	Known
CUL3	CAND1	Known
CUL3	COMMD1	Known
CUL3	DCUN1D1	Known
CUL3	DCUN1D2	Known
CUL3	DCUN1D3	Known

FAM131C	Known
FAM208B	Known
FAM74A4	Known
FAM76B	Known
FAM90A1	Known
FANCA	Known
FARSA	Known
FATE1	Known
FBXL12	Known
FBXO41	Known
FBXW7	Known
FERD3L	Known
FEZ1	Known
FGF12	Known
FKBP4	Known
FKBP5	Known
FLAD1	Known
FLNA	Known
FOLR1	Known
FOS	Known
FOXG1	Known
FOXN3	Known
FOXO1	Known
FOXO3	Known
FOXO4	Known
FRS3	Known
FRYL	Known
FTL	Known
FXR2	Known
FXYD7	Known
FYN	Known
GAB2	Known
GABARAP	Known
GABARAPL1	Known
GABBR1	Known
GABRA1	Known
GATA1	Known
GATA2	Known
GCC1	Known
GCDH	Known
GEM	Known
GIPC1	Known
GLP1R	Known
GLRX3	Known
GLTSCR1L	Known
GLUL	Known
GMCL1	Known

CUL3	DCUN1D4	Known
CUL3	DCUN1D5	Known
CUL3	DYRK4	Known
CUL3	GMCL1	Known
CUL3	KCTD13	Known
CUL3	KCTD5	Known
CUL3	KCTD6	Known
CUL3	KCTD9	Known
CUL3	KLHL12	Known
CUL3	KLHL2	Known
CUL3	KLHL20	Known
CUL3	KLHL3	Known
CUL3	KLHL42	Known
CUL3	KLHL5	Known
CUL3	OLIG2	Known
CUL3	PDIA2	Known
CUL3	RBX1	Known
CUL3	RCBTB1	Known
CUL3	RHOBTB3	Known
CUL3	RNF7	Known
CUL3	SHKBP1	Known
CUL3	UBE2E3	Known
CUL3	UBE2M	Known
CUL3	ZMAT4	Known
DAO	DAOA	Known
DAO	EP300	Known
DAO	PEX5	Known
DAO	PRKAB2	Known
DCN	TNF	Known
DCTN1	DISC1	Known
DCTN1	DTNBP1	Known
DCTN2	DISC1	Known
DDX6	SRPK2	Known
DDX6	TCF4	Known
DHX8	SRPK2	Known
DIS3	MPP6	Known
DISC1	ATF7IP	Known
DISC1	C14orf166	Known
DISC1	CCDC136	Known
DISC1	CCDC141	Known
DISC1	CCDC24	Known
DISC1	CCDC88A	Known
DISC1	CDK5RAP3	Known
DISC1	CEP126	Known
DISC1	CEP57L1	Known
DISC1	CEP63	Known
DISC1	EXOC1	Known

GNA11	Known
GNAI1	Known
GNAI2	Known
GNAI3	Known
GNAO1	Known
GNAQ	Known
GNAZ	Known
GNB1	Known
GNB2L1	Known
GNB5	Known
GNG10	Known
GNPTAB	Known
GOLGA2	Known
GOLGA8EP	Known
GOLGA8F	Known
GOPC	Known
GORASP2	Known
GPRASP2	Known
GPS1	Known
GRASP	Known
GRB10	Known
GRB2	Known
GRIA2	Known
GRIN1	Known
GRIN3A	Known
GRIN3B	Known
GRIP1	Known
GSK3A	Known
GSK3B	Known
GSPT1	Known
GSPT2	Known
GTF2E1	Known
GTF3C5	Known
GUCD1	Known
HAND2	Known
HAT1	Known
HAUS1	Known
HAX1	Known
HBP1	Known
HBS1L	Known
HCN2	Known
HDAC1	Known
HDAC2	Known
HDAC4	Known
HDAC5	Known
HDAC7	Known
HERC2P2	Known

DISC1	EXOC4	Known
DISC1	FBXO41	Known
DISC1	FRYL	Known
DISC1	GNPTAB	Known
DISC1	GPRASP2	Known
DISC1	HERC2P2	Known
DISC1	IFT20	Known
DISC1	KANSL1	Known
DISC1	KCNQ5	Known
DISC1	MEMO1	Known
DISC1	NDE1	Known
DISC1	NDEL1	Known
DISC1	PCNXL4	Known
DISC1	RBSN	Known
DISC1	ROGDI	Known
DISC1	SNX6	Known
DISC1	SPTBN4	Known
DISC1	STX18	Known
DISC1	SYBU	Known
DISC1	TUBB	Known
DLG1	GRIN2A	Known
DLG2	GRIN2A	Known
DLG3	GRIN2A	Known
DLG4	GRIN2A	Known
DLG4	HTR2A	Known
DLG4	NLGN4X	Known
DLGAP2	NLGN4X	Known
DMD	DISC1	Known
DNAJC7	DISC1	Known
DNM2	MPP6	Known
DNMT1	RGS6	Known
DOK1	FES	Known
DPYD	GOPC	Known
DPYD	LXN	Known
DPYSL2	DISC1	Known
DPYSL3	DISC1	Known
DRD2	CADPS	Known
DRD2	CADPS2	Known
DRD2	CLIC6	Known
DRD2	EPB41	Known
DRD2	EPB41L1	Known
DRD2	FLNA	Known
DRD2	GIPC1	Known
DRD2	GNAI2	Known
DRD2	GNAI3	Known
DRD2	GNAZ	Known
DRD2	GRIA2	Known

HGS	Known
HIC1	Known
HIRIP3	Known
HIST2H2AC	Known
HIST2H2BE	Known
HIST2H3C	Known
HIST3H3	Known
HLA-DMB	Known
HMGN3	Known
HMOX1	Known
HNRNPC	Known
HNRNPD	Known
HOMER3	Known
HSF2	Known
HSH2D	Known
HSP90AA1	Known
HSP90AB1	Known
HSPA4	Known
HSPB1	Known
HSPB7	Known
HTRA1	Known
HTT	Known
IBTK	Known
ID1	Known
ID2	Known
ID3	Known
ID4	Known
IFIT3	Known
IFIT5	Known
IFNG	Known
IFT20	Known
IGF1R	Known
IKBKB	Known
IKZF3	Known
IKZF4	Known
IL16	Known
IL4R	Known
ILK	Known
IMMT	Known
IMPDH2	Known
INADL	Known
INPP5K	Known
INS	Known
IPO5	Known
IQSEC1	Known
IRAK1	Known
IRS1	Known

DRD2	KCNJ6	Known
DRD2	KCNJ9	Known
DRD2	NCS1	Known
DRD2	NSF	Known
DRD2	PAWR	Known
DRD2	PPP1R9B	Known
DRD2	SLC6A3	Known
DRD2	SSTR5	Known
DRD3	CLIC6	Known
DRD3	EEF1B2	Known
DRD3	EEF1G	Known
DRD3	EPB41	Known
DRD3	EPB41L1	Known
DRD3	EPB41L2	Known
DRD3	FLNA	Known
DRD3	GIPC1	Known
DRD3	GNAI1	Known
DRD3	GRB2	Known
DRD3	MPDZ	Known
DRD3	NCK1	Known
DRD3	NCS1	Known
DRD3	RDX	Known
DRD3	USP48	Known
DRD4	CLIC6	Known
DRD4	GABRA1	Known
DRD4	GRB2	Known
DRD4	KCNJ9	Known
DRD4	KLHL12	Known
DRD4	NCK1	Known
DRG1	SRPK2	Known
DST	DISC1	Known
DST	DTNBP1	Known
DTNA	DTNBP1	Known
DTNB	DTNBP1	Known
DTNBP1	CCDC153	Known
DTNBP1	CMYA5	Known
DTNBP1	DGCR6L	Known
DTNBP1	HAUS1	Known
DTNBP1	IFT20	Known
DTNBP1	KANSL1	Known
DTNBP1	P4HA3	Known
DTNBP1	SFR1	Known
DTNBP1	SSC5D	Known
DTNBP1	TRIM9	Known
DTNBP1	TXLNB	Known
DYNC1H1	DISC1	Known
DYNLL1	AMBRA1	Known

IRS2	Known
ITGB3	Known
ITPKC	Known
ITPR1	Known
ITPR3	Known
ITSN1	Known
JADE1	Known
JAK1	Known
JAK2	Known
JAK3	Known
JAKMIP2	Known
JUN	Known
JUNB	Known
JUP	Known
KALRN	Known
KANSL1	Known
KAT2B	Known
KAT6A	Known
KCNJ6	Known
KCNJ9	Known
KCNN2	Known
KCNQ5	Known
KCTD13	Known
KCTD14	Known
KCTD5	Known
KCTD6	Known
KCTD9	Known
KEAP1	Known
KHDRBS1	Known
KIAA0408	Known
KIDINS220	Known
KIF3A	Known
KIF3C	Known
KIFAP3	Known
KIFC3	Known
KLC3	Known
KLHL12	Known
KLHL2	Known
KLHL20	Known
KLHL3	Known
KLHL32	Known
KLHL42	Known
KLHL5	Known
KNSTRN	Known
KRIT1	Known
KRT10	Known
KRT31	Known

EED	EPC2	Known
EEF2	DISC1	Known
EFNB1	CUL3	Known
EGF	NRG1	Known
EGFR	FES	Known
EGFR	NRG1	Known
EGFR	PRKD1	Known
EGFR	RGS4	Known
EGR1	NAB2	Known
EGR2	NAB2	Known
EIF3G	MPP6	Known
EIF3H	DISC1	Known
EIF4EBP1	TCF4	Known
EIF5	NRGN	Known
ELOVL7	DTNBP1	Known
EP300	TCF4	Known
EPAS1	PRKD1	Known
EPB41L1	SRPK2	Known
EPHB6	TCF4	Known
EPS15	SNAP91	Known
ERBB2	NRG1	Known
ERBB3	NRG1	Known
ERBB3	RGS4	Known
ERBB4	NRG1	Known
ERCC3	SRPK2	Known
ERN1	SRPK2	Known
ESR1	PAK6	Known
ESR1	TCF20	Known
ETF1	GSPT1	Known
ETF1	GSPT2	Known
ETF1	KEAP1	Known
ETF1	LIG4	Known
ETF1	PPP2CA	Known
EXOC4	DTNBP1	Known
EXOC7	DISC1	Known
EXOC7	DTNBP1	Known
EXOSC10	MPP6	Known
EXOSC2	MPP6	Known
EXOSC7	MPP6	Known
EXOSC8	MPP6	Known
EXOSC9	MPP6	Known
EZH2	EPC2	Known
FAH	TCF4	Known
FES	BCAR1	Known
FES	DPYSL5	Known
FES	FKBP4	Known
FES	FKBP5	Known

KRT40	Known
KRTAP10-3	Known
KRTAP10-8	Known
KRTAP2-4	Known
KRTAP4-12	Known
KRTAP5-9	Known
KTN1	Known
LASP1	Known
LCA5	Known
LCAT	Known
LCE3D	Known
LDLR	Known
LENG1	Known
LGALS14	Known
LGALSL	Known
LIG4	Known
LIMK1	Known
LIN7A	Known
LITAF	Known
LMO1	Known
LMO4	Known
LNPEP	Known
LNX1	Known
LONP1	Known
LOXL4	Known
LRP1	Known
LRP2	Known
LRP8	Known
LRRK2	Known
LRRN4CL	Known
LSM8	Known
LUC7L	Known
LUC7L2	Known
LUC7L3	Known
LUZP4	Known
LXN	Known
LYL1	Known
LYN	Known
LZTR1	Known
MAB21L2	Known
MACF1	Known
MAD2L1	Known
MAD2L2	Known
MAGEB1	Known
MAGOHB	Known
MAML1	Known
MAML2	Known

FES	HSH2D	Known
FES	HSPA4	Known
FES	IL4R	Known
FES	IRS1	Known
FES	IRS2	Known
FES	JAK1	Known
FES	JAK2	Known
FES	JAK3	Known
FES	MDFI	Known
FES	NEDD4	Known
FES	PDE4DIP	Known
FES	PIK3R1	Known
FES	PLXNA1	Known
FES	POT1	Known
FES	PPID	Known
FES	PTGES3	Known
FES	RASA1	Known
FES	RASA3	Known
FES	STAT3	Known
FES	TERF1	Known
FES	TRIM28	Known
FES	ZNF746	Known
FES	ZNF775	Known
FEZ1	DISC1	Known
FGF12	SRPK2	Known
FLNA	TCF4	Known
FOLR1	CUL3	Known
FOS	NFATC3	Known
FOXN3	SRPK2	Known
FTL	MPP6	Known
FYN	GRIN2A	Known
FYN	TNF	Known
GABBR1	RGS4	Known
GEM	TCF4	Known
GIGYF2	WBP11	Known
GLP1R	NRGN	Known
GLTSCR1L	DTNBP1	Known
GLUL	HTR2A	Known
GNA11	HTR2A	Known
GNAI1	HTR2A	Known
GNAI1	RGS4	Known
GNAI2	RGS4	Known
GNAO1	RGS4	Known
GNAQ	HTR2A	Known
GNAQ	RGS4	Known
GNB1	DISC1	Known
GNG10	TCF4	Known

MAML3	Known
MAP1A	Known
MAP2	Known
MAP2K4	Known
MAP2K7	Known
MAP3K11	Known
MAP3K5	Known
MAP3K7	Known
MAP3K8	Known
MAPK14	Known
MAPK3	Known
MAPK8	Known
MAPK8IP1	Known
MAPK8IP2	Known
MAPK9	Known
MAPKAPK2	Known
MAPKAPK5	Known
MAPKBP1	Known
MAPT	Known
MAR10	Known
MAST1	Known
MATN3	Known
MATR3	Known
MAX	Known
MBD3	Known
MBTPS1	Known
MCM7	Known
MCOLN3	Known
MDFI	Known
MDK	Known
MDM2	Known
MDM4	Known
MEMO1	Known
MEN1	Known
METTL1	Known
MFAP1	Known
MID1IP1	Known
MLLT10	Known
MLLT6	Known
MLX	Known
MMP17	Known
MORF4L1	Known
MORN4	Known
MPDZ	Known
MPP3	Known
MPPED1	Known
MRFAP1L1	Known

GOLGA2	PPP1R16B	Known
GPM6A	PAM16	Known
GPM6A	PRKCA	Known
GPS1	CUL3	Known
GRB10	GIGYF2	Known
GRIN1	GRIN2A	Known
GRIN2A	AP1M1	Known
GRIN2A	AP4M1	Known
GRIN2A	ARHGAP32	Known
GRIN2A	CASK	Known
GRIN2A	DLGAP1	Known
GRIN2A	GRIN3A	Known
GRIN2A	GRIN3B	Known
GRIN2A	IL16	Known
GRIN2A	INADL	Known
GRIN2A	NEDD4	Known
GRIN2A	PLCG1	Known
GRIN2A	PRKCA	Known
GRIN2A	PTPN4	Known
GRIN2A	RGS3	Known
GRIN2A	SPTAN1	Known
GRIN2A	SRC	Known
GRM3	GRASP	Known
GRM3	GRIP1	Known
GRM3	PICK1	Known
GRM3	PPM1A	Known
GRM3	SDCBP	Known
GSK3B	AKT3	Known
GTF2E1	SRPK2	Known
HCN2	HCN1	Known
HDAC1	MAD1L1	Known
HDAC1	SATB2	Known
HDAC2	MAD1L1	Known
HIC1	TCF4	Known
HIST3H3	KDM3B	Known
HLA-DMB	CUL3	Known
HNRNPC	SRPK2	Known
HNRNPD	IMMP2L	Known
HSF2	CUL3	Known
HSPB1	PRKD1	Known
HTR2A	ANKS1B	Known
HTR2A	EIF3F	Known
HTR2A	JAK2	Known
HTR2A	MAP1A	Known
HTR2A	MRPL28	Known
HTR2A	NDUFB10	Known
HTR2A	NME3	Known

MRPL10	Known
MRPL28	Known
MRPS6	Known
MS4A2	Known
MSC	Known
MSRB3	Known
MST1R	Known
MT2A	Known
MT2P1	Known
MTA2	Known
MTCP1	Known
MTOR	Known
MUC1	Known
MUC7	Known
MUL1	Known
MVP	Known
MXD1	Known
MYO1A	Known
MYO5B	Known
MYOD1	Known
MYT1L	Known
NBEA	Known
NCAM1	Known
NCK1	Known
NCK2	Known
NCOA4	Known
NCOR2	Known
NCS1	Known
NDC80	Known
NDE1	Known
NDEL1	Known
NDN	Known
NDOR1	Known
NDRG1	Known
NDUFB10	Known
NEBL	Known
NECAP1	Known
NEDD4	Known
NEDD4L	Known
NEDD8	Known
NEDD9	Known
NEFL	Known
NEFM	Known
NEK2	Known
NEK6	Known
NEK8	Known
NELFE	Known

HTR2A	NTRK3	Known
HTR2A	PON2	Known
HTR2A	PPP5C	Known
HTR2A	RPS6KA3	Known
ID1	TCF4	Known
ID2	TCF4	Known
ID3	TCF4	Known
ID4	TCF4	Known
IFNG	TNF	Known
IGF1R	PRKD1	Known
IKZF3	PPP1R16B	Known
IMMT	DISC1	Known
INS	TCF4	Known
IPO5	DTNBP1	Known
ITSN1	DISC1	Known
JUN	PRKD1	Known
JUN	TCF20	Known
JUN	TCF4	Known
JUP	TCF4	Known
KALRN	DISC1	Known
KALRN	DTNBP1	Known
KCNN2	SRPK2	Known
KDM3B	ZNF512B	Known
KHDRBS1	MPP6	Known
KIAA0408	DTNBP1	Known
KIF3A	DISC1	Known
KIF3C	DISC1	Known
KIFAP3	DISC1	Known
KIFC3	DTNBP1	Known
KRIT1	RGS4	Known
LASP1	TCF4	Known
LIN7A	MPP6	Known
LMO1	TCF4	Known
LNPEP	TCF4	Known
LYL1	TCF4	Known
LYN	TNF	Known
MACF1	DISC1	Known
MACF1	DTNBP1	Known
MAD1L1	AMOTL2	Known
MAD1L1	BAG5	Known
MAD1L1	CCDC94	Known
MAD1L1	CCHCR1	Known
MAD1L1	CWF19L2	Known
MAD1L1	FAM131C	Known
MAD1L1	HAUS1	Known
MAD1L1	LGALSL	Known
MAD1L1	NDC80	Known

NEU4	Known
NEUROD1	Known
NEUROG1	Known
NF2	Known
NFKB1	Known
NFKBIA	Known
NGLY1	Known
NINL	Known
NME3	Known
NME7	Known
NMUR2	Known
NOL9	Known
NONO	Known
NOP16	Known
NOS3	Known
NOTCH1	Known
NOTCH2NL	Known
NPM2	Known
NR2C2AP	Known
NR2E1	Known
NR2F1	Known
NR2F2	Known
NR4A1	Known
NSF	Known
NSMCE4A	Known
NSRP1	Known
NTF3	Known
NTRK2	Known
NTRK3	Known
NUDT10	Known
NUF2	Known
NUP160	Known
NUP50	Known
NXT2	Known
OCEL1	Known
OGT	Known
OLFM1	Known
OLIG2	Known
ORC4	Known
OSGIN1	Known
OTUD4	Known
P4HA3	Known
PABPC4	Known
PAFAH1B1	Known
PAK1	Known
PAK4	Known
PAM16	Known

MAD1L1	NEBL	Known
MAD1L1	NINL	Known
MAD1L1	RNF8	Known
MAD1L1	SIN3A	Known
MAD1L1	SIN3B	Known
MAD1L1	SPATA2	Known
MAD1L1	TEX11	Known
MAD1L1	TRIM29	Known
MAD1L1	TSHZ3	Known
MAD1L1	TUBGCP4	Known
MAD1L1	USP15	Known
MAD1L1	ZSCAN32	Known
MAD2L1	MAD1L1	Known
MAGEB1	SRPK2	Known
MAN2A1	SEC23B	Known
MAP1A	DISC1	Known
MAP2K7	VRK2	Known
MAP3K7	VRK2	Known
MAPK14	PAK6	Known
MAPK14	TCF20	Known
MATN3	TCF4	Known
MATR3	DISC1	Known
MAX	MAD1L1	Known
MBD3	BCL11B	Known
MCM7	NAB2	Known
MDFI	ADAMTSL3	Known
MDFI	NAB2	Known
MDK	SRPK2	Known
MDM2	PAK6	Known
MEN1	TCF4	Known
MFAP1	MAD1L1	Known
MLLT10	DISC1	Known
MLLT6	TCF4	Known
MLX	MAD1L1	Known
MMP17	TNF	Known
MPP3	SRPK2	Known
MPP6	ARHGAP18	Known
MPP6	DYNLRB1	Known
MPP6	EXOSC4	Known
MPP6	EXOSC5	Known
MPP6	EXOSC6	Known
MPP6	KNSTRN	Known
MPPED1	DISC1	Known
MT2A	PRKD1	Known
MT2P1	MPP6	Known
MTA2	BCL11B	Known
MTA2	SATB2	Known

PARK2	Known
PARN	Known
PARP1	Known
PATE1	Known
PAWR	Known
PBLD	Known
PCBD1	Known
PCMT1	Known
PCNT	Known
PCNXL4	Known
PDCD4	Known
PDE3B	Known
PDE4B	Known
PDE4DIP	Known
PDE7B	Known
PDIA2	Known
PDIK1L	Known
PDK2	Known
PDPK1	Known
PDZK1	Known
PEA15	Known
PELI1	Known
PEX5	Known
PFKFB1	Known
PFKFB2	Known
PGK1	Known
PHB2	Known
PHC2	Known
PHF1	Known
PHOSPHO2	Known
PI4K2B	Known
PIAS1	Known
PIAS2	Known
PICK1	Known
PIK3R1	Known
PIN1	Known
PKN2	Known
PLCB1	Known
PLCG1	Known
PLCG2	Known
PLEKHA6	Known
PLEKHN1	Known
PLEKHO1	Known
PLN	Known
PLSCR1	Known
PLTP	Known
PLXNA1	Known

MTHFR	LSM8	Known
MTOR	AMBRA1	Known
MUC1	GALNT10	Known
MUC7	GALNT10	Known
MVP	DISC1	Known
MYO1A	DISC1	Known
MYO5B	TCF4	Known
MYOD1	TCF4	Known
MYT1L	DISC1	Known
NAB2	CCDC33	Known
NAB2	CCHCR1	Known
NAB2	CWF19L2	Known
NAB2	DGCR6	Known
NAB2	GCC1	Known
NAB2	JAKMIP2	Known
NAB2	KRT40	Known
NAB2	KRTAP10-3	Known
NAB2	KRTAP10-8	Known
NAB2	LENG1	Known
NAB2	MAPK3	Known
NAB2	MORF4L1	Known
NAB2	NEDD9	Known
NAB2	NME7	Known
NAB2	NOTCH2NL	Known
NAB2	PHF1	Known
NAB2	PHOSPHO2	Known
NAB2	PIN1	Known
NAB2	RBPMS	Known
NAB2	RFC5	Known
NAB2	SCNM1	Known
NAB2	SUMO1P1	Known
NAB2	TCEANC	Known
NAB2	TCHP	Known
NAB2	TGIF2LY	Known
NAB2	TTC19	Known
NAB2	ZBED8	Known
NAB2	ZMYND19	Known
NBEA	DTNBP1	Known
NDN	DTNBP1	Known
NDN	MPP6	Known
NEDD4	AKT3	Known
NEDD4	PRRG2	Known
NEDD4	SLC6A3	Known
NEDD4L	CYP26B1	Known
NEDD8	CUL3	Known
NEFL	PPP1R16B	Known
NEFM	DISC1	Known

POLB	Known
POLR1A	Known
POLR1C	Known
PON2	Known
POT1	Known
POU2F1	Known
PPARD	Known
PPARGC1B	Known
PPIA	Known
PPID	Known
PPIL1	Known
PPL	Known
PPM1A	Known
PPM1E	Known
PPP1CA	Known
PPP1CC	Known
PPP1R14A	Known
PPP1R18	Known
PPP1R9B	Known
PPP2CA	Known
PPP2R1A	Known
PPP2R4	Known
PPP4R1	Known
PPP5C	Known
PRAM1	Known
PRDX2	Known
PRG2	Known
PRKAA1	Known
PRKAA2	Known
PRKAB2	Known
PRKACA	Known
PRKCA	Known
PRKCB	Known
PRKCD	Known
PRKCE	Known
PRKCG	Known
PRKCH	Known
PRKCQ	Known
PRKCZ	Known
PRKDC	Known
PRNP	Known
PRPF38A	Known
PRPF40A	Known
PRRC2A	Known
PRRC2B	Known
PRTN3	Known
PSEN1	Known

NEK2	MAD1L1	Known
NEK6	PAK6	Known
NEUROD1	TCF4	Known
NEUROG1	TCF4	Known
NFATC3	MAPK8	Known
NFATC3	MAPK9	Known
NFATC3	TTF1	Known
NFKB1	MPP6	Known
NFKBIA	TNF	Known
NONO	MAD1L1	Known
NOTCH4	DLL4	Known
NOTCH4	EGFL7	Known
NOTCH4	FBXW7	Known
NOTCH4	MAML1	Known
NOTCH4	MAML2	Known
NOTCH4	MAML3	Known
NOTCH4	PSEN1	Known
NOTCH4	PSEN2	Known
NOTCH4	TCEB1	Known
NR2F1	BCL11B	Known
NR2F2	BCL11B	Known
NR4A1	TCF4	Known
NRG1	ADAM19	Known
NRG1	IKZF4	Known
NRG1	LIMK1	Known
NRGN	PRKCA	Known
NRGN	PRKCB	Known
NRGN	PRKCG	Known
NUF2	DTNBP1	Known
NUP160	DISC1	Known
OGT	SNAP91	Known
OLFM1	DISC1	Known
ORC4	TCF4	Known
PAFAH1B1	DISC1	Known
PAK6	RHOJ	Known
PARK2	TCF4	Known
PARN	MPP6	Known
PARP1	TCF4	Known
PCNT	DISC1	Known
PDE4B	DISC1	Known
PDPK1	AKT3	Known
PGK1	DISC1	Known
PHC2	PPP1R16B	Known
PIAS1	SATB2	Known
PIN1	TCF4	Known
PLCG1	PRKD1	Known
PLCG1	SNAP91	Known

PSEN2	Known
PSMA1	Known
PSMA3	Known
PSME3	Known
PSMG2	Known
PTAFR	Known
PTBP1	Known
PTEN	Known
PTF1A	Known
PTGER3	Known
PTGES3	Known
PTGS1	Known
PTGS2	Known
PTK2B	Known
PTPN1	Known
PTPN4	Known
RAB11A	Known
RAB3D	Known
RAB3IL1	Known
RAB41	Known
RABGAP1	Known
RABIF	Known
RAC1	Known
RAD21	Known
RAF1	Known
RANBP9	Known
RARA	Known
RASA1	Known
RASA3	Known
RASSF7	Known
RBBP4	Known
RBBP7	Known
RBFOX2	Known
RBM12	Known
RBM15	Known
RBM23	Known
RBM39	Known
RBM8A	Known
RBPJ	Known
RBPMS	Known
RBSN	Known
RBX1	Known
RC3H1	Known
RCBTB1	Known
RCBTB2	Known
RDX	Known
RECK	Known

PLCG2	PRKD1	Known
POLB	SRPK2	Known
POU2F1	TNF	Known
PPARD	SHMT2	Known
PPIA	SRPK2	Known
PPIA	TCF4	Known
PPM1E	DISC1	Known
PPP1CC	SMG6	Known
PPP1R16B	CDCA7L	Known
PPP1R16B	CEP55	Known
PPP1R16B	CEP70	Known
PPP1R16B	FAM208B	Known
PPP1R16B	KCTD9	Known
PPP3CC	CABIN1	Known
PPP3CC	HAX1	Known
PPP3CC	ITPKC	Known
PPP3CC	SDC2	Known
PPP3CC	TGFBR1	Known
PPP3CC	THAP7	Known
PPP4R1	DISC1	Known
PPP5C	DISC1	Known
PRKAA1	SRPK2	Known
PRKAA2	TCF4	Known
PRKAB2	TCF4	Known
PRKCD	AKT3	Known
PRKCE	PRKD1	Known
PRKCH	PRKD1	Known
PRKCZ	AKT3	Known
PRKD1	ADAP1	Known
PRKD1	AKAP13	Known
PRKD1	COPS2	Known
PRKD1	COPS5	Known
PRKD1	COPS7A	Known
PRKD1	GNB2L1	Known
PRKD1	HDAC5	Known
PRKD1	HDAC7	Known
PRKD1	IBTK	Known
PRKD1	KIDINS220	Known
PRKD1	MAPK8	Known
PRKD1	MAPK9	Known
PRKD1	PPP1R14A	Known
PRKD1	SRC	Known
PRKD1	SYK	Known
PRKD1	TP53	Known
PRKD1	YWHAQ	Known
PRKD1	YWHAZ	Known
PRRG2	FATE1	Known

RELA	Known
REM1	Known
REXO1L6P	Known
RFC5	Known
RGCC	Known
RGS2	Known
RGS3	Known
RHEB	Known
RHEBL1	Known
RHOBTB3	Known
RHOJ	Known
RICTOR	Known
RIMS1	Known
RIPK1	Known
RIT1	Known
RIT2	Known
RNF11	Known
RNF115	Known
RNF138	Known
RNF2	Known
RNF32	Known
RNF4	Known
RNF7	Known
RNF8	Known
RNPS1	Known
ROGDI	Known
RPL22	Known
RPL39L	Known
RPL4	Known
RPL41	Known
RPS20	Known
RPS6KA3	Known
RPS6KB1	Known
RQCD1	Known
RSPH14	Known
RSRC1	Known
RSRC2	Known
RSRP1	Known
RTF1	Known
RUNX1T1	Known
RWDD2B	Known
RYR2	Known
S100A1	Known
S1PR1	Known
SCARB1	Known
SCNM1	Known
SCOC	Known

PRTN3	TNF	Known
PSEN1	TCF4	Known
PSMA1	MAD1L1	Known
PSMA1	TCF4	Known
PSMA3	SRPK2	Known
PSME3	DTNBP1	Known
PTAFR	RGS4	Known
PTBP1	SRPK2	Known
PTGER3	TCF4	Known
PTGIS	PTGS1	Known
PTGIS	PTGS2	Known
PTK2B	GRIN2A	Known
RAB11A	DTNBP1	Known
RAB3IL1	SATB2	Known
RABGAP1	DISC1	Known
RABIF	TCF4	Known
RAD21	DISC1	Known
RAD21	STAG1	Known
RANBP9	DISC1	Known
RASSF7	DISC1	Known
RBBP4	BCL11B	Known
RBBP7	BCL11B	Known
RBM12	GIGYF2	Known
RBPJ	NOTCH4	Known
RBSN	DTNBP1	Known
RCBTB2	CUL3	Known
RELA	TCF4	Known
RERE	ALG13	Known
RERE	ATN1	Known
RERE	CBFA2T2	Known
RERE	ECM1	Known
RERE	EFEMP1	Known
RERE	EFEMP2	Known
RERE	EHMT2	Known
RERE	HIST2H3C	Known
RERE	KAT6A	Known
RERE	KRTAP4-12	Known
RERE	LZTR1	Known
RERE	NR2E1	Known
RERE	PLSCR1	Known
RERE	PRRC2A	Known
RERE	PRRC2B	Known
RERE	PSMA3	Known
RERE	RBFOX2	Known
RERE	TRIM22	Known
RERE	TRIP6	Known
RERE	ZMYND8	Known

SDC2	Known
SDCBP	Known
SDR42E1	Known
SEC14L4	Known
SEC23B	Known
SEMA3B	Known
SETDB1	Known
SF3B4	Known
SFR1	Known
SH2B2	Known
SH3BP5	Known
SH3RF1	Known
SHKBP1	Known
SIGLEC6	Known
SIN3A	Known
SIN3B	Known
SIRT1	Known
SIRT6	Known
SKI	Known
SKIV2L2	Known
SKP2	Known
SLAIN2	Known
SLC39A13	Known
SLC4A1AP	Known
SLC9A3R1	Known
SMAD1	Known
SMAD2	Known
SMAD3	Known
SMAD4	Known
SMAD7	Known
SMAD9	Known
SMARCA4	Known
SMARCB1	Known
SMARCC1	Known
SMARCE1	Known
SMC2	Known
SMC3	Known
SMCP	Known
SMG5	Known
SMOC1	Known
SNAPIN	Known
SNCA	Known
SNIP1	Known
SNRNP27	Known
SNRNP35	Known
SNRNP70	Known
SNRPB	Known

RGS4	COPB2	Known
RGS4	PLCB1	Known
RGS6	DMAP1	Known
RGS6	GNB5	Known
RGS6	STMN2	Known
RIT1	CUL3	Known
RIT1	SRPK2	Known
RIT2	SRPK2	Known
RNF2	AMBRA1	Known
RNF4	SRPK2	Known
RNF4	TCF20	Known
RPL22	SRPK2	Known
RPL41	SRPK2	Known
RPS20	MPP6	Known
RQCD1	GIGYF2	Known
RUNX1T1	TCF4	Known
SDCBP	DTNBP1	Known
SEMA3B	PAK6	Known
SF3B4	PPP1R16B	Known
SH3BP5	DISC1	Known
SHMT2	ARL6IP1	Known
SHMT2	CMTM5	Known
SHMT2	NDRG1	Known
SIGLEC6	TCF4	Known
SKIV2L2	MPP6	Known
SLC6A3	GNB2L1	Known
SLC6A3	PICK1	Known
SLC6A3	SNCA	Known
SLC6A3	SNTA1	Known
SLC6A3	STX1A	Known
SLC6A3	TGFB1I1	Known
SLC6A4	STX1A	Known
SMAD1	TCF20	Known
SMAD2	NOTCH4	Known
SMAD2	STAG1	Known
SMAD3	NOTCH4	Known
SMAD4	NOTCH4	Known
SMAD9	STAG1	Known
SMARCA4	MPP6	Known
SMARCE1	DISC1	Known
SMC2	DISC1	Known
SMC3	DISC1	Known
SMCP	TCF4	Known
SMG6	SMG5	Known
SNAP91	NECAP1	Known
SNAP91	TRAF3IP1	Known
SNAP91	TUBA1B	Known

SNTA1	Known
SNURF	Known
SNX2	Known
SNX6	Known
SNX9	Known
SORBS2	Known
SORBS3	Known
SORT1	Known
SP1	Known
SPAG8	Known
SPARCL1	Known
SPATA2	Known
SPATC1L	Known
SPG21	Known
SPP1	Known
SPTAN1	Known
SPTBN1	Known
SPTBN4	Known
SRC	Known
SREK1	Known
SRGAP2	Known
SRGAP3	Known
SRI	Known
SRRM1	Known
SRSF1	Known
SRSF12	Known
SRSF2	Known
SRSF3	Known
SRSF5	Known
SRSF7	Known
SRSF8	Known
SRSF9	Known
SSC5D	Known
SSTR5	Known
ST13	Known
STAG2	Known
STAMBPL1	Known
STAT1	Known
STAT3	Known
STK16	Known
STK3	Known
STK4	Known
STMN2	Known
STON1	Known
STX11	Known
STX18	Known
STX1A	Known

SNAPIN	DTNBP1	Known
SNRNP70	SRPK2	Known
SNRPB	GIGYF2	Known
SNX2	PAK6	Known
SNX9	MPP6	Known
SP1	BCL11B	Known
SPAG8	KDM3B	Known
SPARCL1	DISC1	Known
SPP1	ZNF804A	Known
SPTAN1	DISC1	Known
SPTBN1	DISC1	Known
SRGAP2	DISC1	Known
SRGAP3	DISC1	Known
SRGAP3	DTNBP1	Known
SRI	SRPK2	Known
SRPK2	MAR10	Known
SRPK2	ACIN1	Known
SRPK2	ADGRG5	Known
SRPK2	AHCYL1	Known
SRPK2	ALKBH3	Known
SRPK2	AMMECR1L	Known
SRPK2	APOBEC3C	Known
SRPK2	ARGLU1	Known
SRPK2	ARHGAP12	Known
SRPK2	ARL6IP4	Known
SRPK2	ARPC1B	Known
SRPK2	ATAT1	Known
SRPK2	AURKA	Known
SRPK2	BAIAP2	Known
SRPK2	BRD3	Known
SRPK2	C16orf78	Known
SRPK2	C17orf85	Known
SRPK2	C18orf25	Known
SRPK2	C1orf35	Known
SRPK2	C4orf46	Known
SRPK2	CACNG2	Known
SRPK2	CAND1	Known
SRPK2	CASK	Known
SRPK2	CBLN4	Known
SRPK2	CBX5	Known
SRPK2	CCDC28A	Known
SRPK2	CCDC97	Known
SRPK2	CCM2	Known
SRPK2	CDC42EP4	Known
SRPK2	CHERP	Known
SRPK2	CHTOP	Known
SRPK2	CLDN19	Known

SUMO1P1	Known
SUMO2	Known
SUMO3	Known
SUPT16H	Known
SUV39H1	Known
SVIL	Known
SYBU	Known
SYK	Known
SYNE1	Known
SZT2	Known
TADA2A	Known
TAF7L	Known
TAL1	Known
TAL2	Known
TAPBPL	Known
TBC1D4	Known
TCEA2	Known
TCEANC	Known
TCEB1	Known
TCF3	Known
TCHP	Known
TCL1A	Known
TCL1B	Known
TCL6	Known
TCOF1	Known
TCP1	Known
TDP2	Known
TERF1	Known
TERF2IP	Known
TERT	Known
TEX11	Known
TFIP11	Known
TGFB1I1	Known
TGFBR1	Known
TGIF2LY	Known
THAP7	Known
THEM4	Known
THOP1	Known
TIAM2	Known
TLE1	Known
TLE4	Known
TMEM213	Known
TNFAIP1	Known
TNFAIP3	Known
TNFRSF10C	Known
TNFRSF1A	Known
TNFRSF1B	Known

SRPK2	CSAG1	Known
SRPK2	CTNNBL1	Known
SRPK2	DAW1	Known
SRPK2	DBF4B	Known
SRPK2	DDX21	Known
SRPK2	DDX46	Known
SRPK2	DNAJC8	Known
SRPK2	DRAP1	Known
SRPK2	EGLN2	Known
SRPK2	EIF1AY	Known
SRPK2	ENOX2	Known
SRPK2	EPB41L4A	Known
SRPK2	EPS8L3	Known
SRPK2	EVI5L	Known
SRPK2	FAM76B	Known
SRPK2	FAM90A1	Known
SRPK2	FXR2	Known
SRPK2	GABARAP	Known
SRPK2	GABARAPL1	Known
SRPK2	HBS1L	Known
SRPK2	HIRIP3	Known
SRPK2	HMGN3	Known
SRPK2	HOMER3	Known
SRPK2	IFIT5	Known
SRPK2	LCA5	Known
SRPK2	LCE3D	Known
SRPK2	LRRN4CL	Known
SRPK2	LUC7L	Known
SRPK2	LUC7L2	Known
SRPK2	LUC7L3	Known
SRPK2	LUZP4	Known
SRPK2	MAB21L2	Known
SRPK2	MAPKAPK5	Known
SRPK2	MCOLN3	Known
SRPK2	MRPS6	Known
SRPK2	NCK2	Known
SRPK2	NELFE	Known
SRPK2	NGLY1	Known
SRPK2	NOP16	Known
SRPK2	NPM2	Known
SRPK2	NSMCE4A	Known
SRPK2	NSRP1	Known
SRPK2	NUP50	Known
SRPK2	NXT2	Known
SRPK2	OCEL1	Known
SRPK2	PABPC4	Known
SRPK2	PAK4	Known

TNFSF11	Known
TNIK	Known
TNIP2	Known
TNKS	Known
TOPBP1	Known
TP53	Known
TPD52	Known
TPD52L1	Known
TPM1	Known
TPM3	Known
TPR	Known
TRA2A	Known
TRA2B	Known
TRADD	Known
TRAF2	Known
TRAF3IP1	Known
TRAF6	Known
TRAPPC2L	Known
TRIB3	Known
TRIM13	Known
TRIM2	Known
TRIM22	Known
TRIM27	Known
TRIM28	Known
TRIM29	Known
TRIM32	Known
TRIM41	Known
TRIM9	Known
TRIO	Known
TRIP13	Known
TRIP6	Known
TSC1	Known
TSC2	Known
TSC22D3	Known
TSHZ3	Known
TSSC4	Known
TSSK3	Known
TTC19	Known
TTC3	Known
TTF1	Known
TTN	Known
TUBA1B	Known
TUBB	Known
TUBB2A	Known
TUBGCP4	Known
TWIST1	Known
TWIST2	Known

SRPK2	PDE7B	Known
SRPK2	PELI1	Known
SRPK2	PPIL1	Known
SRPK2	PRPF38A	Known
SRPK2	PRPF40A	Known
SRPK2	RBM15	Known
SRPK2	RBM23	Known
SRPK2	RBM39	Known
SRPK2	RBM8A	Known
SRPK2	RNPS1	Known
SRPK2	RPL39L	Known
SRPK2	RSRC1	Known
SRPK2	RSRC2	Known
SRPK2	RSRP1	Known
SRPK2	RTF1	Known
SRPK2	RWDD2B	Known
SRPK2	SDR42E1	Known
SRPK2	SEC23B	Known
SRPK2	SLAIN2	Known
SRPK2	SNIP1	Known
SRPK2	SNRNP27	Known
SRPK2	SNRNP35	Known
SRPK2	SNURF	Known
SRPK2	SREK1	Known
SRPK2	SRRM1	Known
SRPK2	SRSF12	Known
SRPK2	SRSF8	Known
SRPK2	SRSF9	Known
SRPK2	STON1	Known
SRPK2	SUPT16H	Known
SRPK2	TAF7L	Known
SRPK2	TCOF1	Known
SRPK2	TDP2	Known
SRPK2	TLE4	Known
SRPK2	TNFRSF10C	Known
SRPK2	TNIP2	Known
SRPK2	TPD52	Known
SRPK2	TRA2A	Known
SRPK2	TRIM41	Known
SRPK2	TTN	Known
SRPK2	U2AF1	Known
SRPK2	U2AF2	Known
SRPK2	UBD	Known
SRPK2	UBE2E2	Known
SRPK2	VAV2	Known
SRPK2	VIMP	Known
SRPK2	VRK1	Known

TXLNB	Known
TXNL4B	Known
TYRO3	Known
U2AF1	Known
U2AF2	Known
UBC	Known
UBD	Known
UBE2D1	Known
UBE2D2	Known
UBE2D3	Known
UBE2E1	Known
UBE2E2	Known
UBE2E3	Known
UBE2M	Known
UBE2S	Known
UBQLN1	Known
UBTF	Known
USP15	Known
USP4	Known
USP48	Known
UTP23	Known
UTRN	Known
UXS1	Known
VAV2	Known
VEGFA	Known
VHL	Known
VIM	Known
VIMP	Known
VKORC1	Known
VLDLR	Known
VPS28	Known
VRK1	Known
WBP11	Known
WDR5	Known
WFDC5	Known
WNK1	Known
WNK4	Known
WNT7B	Known
XIAP	Known
XPNPEP1	Known
XRCC5	Known
XRCC6	Known
XRN2	Known
YAP1	Known
YBX1	Known
YPEL2	Known
YPEL3	Known

SRPK2	WDR5	Known
SRPK2	YPEL2	Known
SRPK2	YPEL3	Known
SRPK2	YTHDC1	Known
SRPK2	YWHAB	Known
SRPK2	ZMAT4	Known
SRPK2	ZRANB2	Known
SRPK2	ZRSR2	Known
SRPK2	ZSCAN9	Known
SRSF1	SRPK2	Known
SRSF2	TCF4	Known
SRSF3	SRPK2	Known
SRSF5	SRPK2	Known
SRSF7	SRPK2	Known
STAG1	STAG2	Known
STAG1	WFDC5	Known
SUMO2	CUL3	Known
SUMO3	CUL3	Known
SUV39H1	BCL11B	Known
SVIL	MPHOSPH9	Known
SYBU	DTNBP1	Known
SYNE1	DISC1	Known
SYNE1	DTNBP1	Known
TADA2A	PPP1R16B	Known
TAL1	TCF4	Known
TAL2	TCF4	Known
TCEA2	TCF4	Known
TCEB1	CUL3	Known
TCF20	HIST2H2AC	Known
TCF20	HIST2H2BE	Known
TCF4	ARHGEF1	Known
TCF4	ARMC7	Known
TCF4	ASCL3	Known
TCF4	ASCL4	Known
TCF4	ASPSCR1	Known
TCF4	BCAS2	Known
TCF4	BZRAP1	Known
TCF4	C19orf66	Known
TCF4	C1orf109	Known
TCF4	C9orf171	Known
TCF4	CABP5	Known
TCF4	CCDC101	Known
TCF4	CDC73	Known
TCF4	CHAF1A	Known
TCF4	CHCHD2	Known
TCF4	CHIC2	Known
TCF4	DEF6	Known

YTHDC1	Known
YWHAB	Known
YWHAE	Known
YWHAG	Known
YWHAQ	Known
YWHAZ	Known
ZBED8	Known
ZDHHC17	Known
ZDHHC24	Known
ZFP36L1	Known
ZHX1	Known
ZMAT4	Known
ZMYND19	Known
ZMYND8	Known
ZNF124	Known
ZNF197	Known
ZNF205-AS1	Known
ZNF365	Known
ZNF417	Known
ZNF490	Known
ZNF512B	Known
ZNF558	Known
ZNF587	Known
ZNF746	Known
ZNF775	Known
ZRANB2	Known
ZRSR2	Known
ZSCAN32	Known
ZSCAN9	Known

TCF4	DGCR6	Known
TCF4	DMRT3	Known
TCF4	EFHC1	Known
TCF4	EIF4E2	Known
TCF4	EPB41L3	Known
TCF4	EXOSC1	Known
TCF4	FAM124B	Known
TCF4	FAM74A4	Known
TCF4	FERD3L	Known
TCF4	FLAD1	Known
TCF4	FRS3	Known
TCF4	GLRX3	Known
TCF4	GOLGA8EP	Known
TCF4	GOLGA8F	Known
TCF4	GORASP2	Known
TCF4	GTF3C5	Known
TCF4	GUCD1	Known
TCF4	HAND2	Known
TCF4	HAT1	Known
TCF4	HBP1	Known
TCF4	HSPB7	Known
TCF4	KCTD14	Known
TCF4	KLC3	Known
TCF4	KLHL32	Known
TCF4	LENG1	Known
TCF4	LGALS14	Known
TCF4	LMO4	Known
TCF4	MAD2L2	Known
TCF4	MAGOHB	Known
TCF4	MAPKBP1	Known
TCF4	MEMO1	Known
TCF4	MORN4	Known
TCF4	MRFAP1L1	Known
TCF4	MRPL10	Known
TCF4	MSC	Known
TCF4	MSRB3	Known
TCF4	MVP	Known
TCF4	NCK2	Known
TCF4	NDOR1	Known
TCF4	NEK6	Known
TCF4	NEK8	Known
TCF4	NEU4	Known
TCF4	NME7	Known
TCF4	NMUR2	Known
TCF4	NOL9	Known
TCF4	NR2C2AP	Known
TCF4	NUDT10	Known

TCF4	OSGIN1	Known
TCF4	OTUD4	Known
TCF4	PATE1	Known
TCF4	PBLD	Known
TCF4	PLEKHN1	Known
TCF4	POLR1A	Known
TCF4	POLR1C	Known
TCF4	PPIL1	Known
TCF4	PPP1R18	Known
TCF4	PSMG2	Known
TCF4	PTF1A	Known
TCF4	RAB41	Known
TCF4	RECK	Known
TCF4	REXO1L6P	Known
TCF4	RNF138	Known
TCF4	RSPH14	Known
TCF4	SCOC	Known
TCF4	SEC14L4	Known
TCF4	SLC39A13	Known
TCF4	SLC4A1AP	Known
TCF4	SMOC1	Known
TCF4	SPATC1L	Known
TCF4	SPG21	Known
TCF4	STAMBPL1	Known
TCF4	STK16	Known
TCF4	STX11	Known
TCF4	SZT2	Known
TCF4	TAPBPL	Known
TCF4	TCF3	Known
TCF4	TCL1A	Known
TCF4	TLE1	Known
TCF4	TMEM213	Known
TCF4	TP53	Known
TCF4	TRAPPC2L	Known
TCF4	TSSC4	Known
TCF4	TSSK3	Known
TCF4	TWIST1	Known
TCF4	TWIST2	Known
TCF4	TXNL4B	Known
TCF4	USP4	Known
TCF4	UTP23	Known
TCF4	VPS28	Known
TCF4	XRCC5	Known
TCF4	ZDHHC24	Known
TCF4	ZNF124	Known
TCF4	ZNF205-AS1	Known
TCF4	ZNF417	Known

TCF4	ZNF587	Known
TCL1A	AKT3	Known
TCP1	VRK2	Known
TERT	SMG6	Known
TFIP11	DISC1	Known
THOP1	MPP6	Known
TIAM2	DISC1	Known
TNF	ADAM9	Known
TNF	PDIK1L	Known
TNF	RC3H1	Known
TNF	RIPK1	Known
TNF	TNFAIP3	Known
TNF	TNFRSF1A	Known
TNF	TNFRSF1B	Known
TNF	TRADD	Known
TNF	TRAF2	Known
TNF	YWHAQ	Known
TNFAIP1	CUL3	Known
TNIK	DISC1	Known
TNKS	DISC1	Known
TPD52L1	PAK6	Known
TPM1	MAD1L1	Known
TPM3	MAD1L1	Known
TPR	MAD1L1	Known
TRA2B	SRPK2	Known
TRAF3IP1	DISC1	Known
TRAF3IP1	DTNBP1	Known
TRAF6	OTUD7B	Known
TRIM2	DTNBP1	Known
TRIM27	DTNBP1	Known
TRIM32	DTNBP1	Known
TRIO	DISC1	Known
TSC2	AKT3	Known
TSC22D3	MAD1L1	Known
TUBB2A	DISC1	Known
UBC	AMBRA1	Known
UBC	HCN1	Known
UBC	MAD1L1	Known
UBC	MPHOSPH9	Known
UBC	OTUD7B	Known
UBE2D1	CUL3	Known
UBE2D2	CUL3	Known
UBE2D3	AMBRA1	Known
UBE2E1	CUL3	Known
UBE2E2	CUL3	Known
UBE2S	OTUD7B	Known
UBTF	MAD1L1	Known

UTRN	DISC1	Known
VHL	DGKI	Known
VRK2	CCT2	Known
VRK2	CCT4	Known
VRK2	LNX1	Known
VRK2	MAPK8IP1	Known
VRK2	MAPK8IP2	Known
VRK2	RBX1	Known
WNT7B	CUL3	Known
XPNPEP1	DISC1	Known
XRCC6	TCF4	Known
XRN2	DISC1	Known
YBX1	SLC6A4	Known
YWHAE	DISC1	Known
YWHAG	DISC1	Known
YWHAG	MPHOSPH9	Known
YWHAQ	DISC1	Known
YWHAZ	DISC1	Known
YWHAZ	MPHOSPH9	Known
ZDHHC17	OTUD7B	Known
ZDHHC17	TMTC1	Known
ZNF197	DISC1	Known
ZNF365	DISC1	Known
ZNF490	DTNBP1	Known
